# UV induces codirectional replication–transcription conflicts and an alternative DnaA-dependent replication origin in the *rnhAB* mutants of *Escherichiacoli*

**DOI:** 10.1093/nar/gkaf282

**Published:** 2025-04-16

**Authors:** Elena A Kouzminova, Glen E Cronan, Andrei Kuzminov

**Affiliations:** Department of Microbiology, University of Illinois at Urbana-Champaign, Urbana, IL 61801, United States; Department of Microbiology, University of Illinois at Urbana-Champaign, Urbana, IL 61801, United States; Department of Microbiology, University of Illinois at Urbana-Champaign, Urbana, IL 61801, United States

## Abstract

The *rnhAB* mutants of *Escherichia coli* lacking both RNase H enzymes are unexpectedly UV-sensitive, being unable to restore normal levels of post-UV replication. Examining patterns of chromosomal replication in the *rnhAB* mutants after UV could identify the problem sites. We show that normal *rnhA (B)* mutant replication initiates at three distinct *oriK* areas in the origin macrodomain, none of them coinciding with *oriC* proper, the dominant origin being some 400 kb away. Interestingly, initiation after UV switches to the DnaA-dependent *oriK* closest to *oriC* and continues from there until the growth replication pattern is restored, like in the *rnhA* single mutants. However, in the *rnhAB* double mutant, post-UV forks initiated at the new origin have difficulty reaching the terminus, with the major stalling sites at the *rrn* operons. In the *rnhAB recBC* mutants, additionally deficient in linear DNA degradation/repair, post-UV replication forks cannot traverse the origin-distal ribosomal RNA operons, *rrnG* and *rrnH*, showing that restoration of disintegrated replication forks is essential for replication in the *rnhAB* mutant. In contrast, the rnhAB rpoB* mutant, in which transcription complexes are unstable, is UV-resistant and resumes normal replication even faster than WT cells, indicating that the *rnhAB* mutants suffer from UV-induced replication–transcription conflicts.

## Graphical Abstract

**Graphical Abstract:**
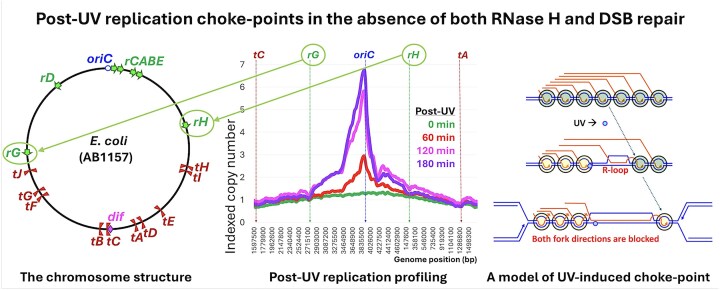

## Introduction

Among the natural DNA-damaging conditions, UV light is unique in delivering well-defined DNA lesions, pyrimidine dimers (PDs) [1], that block progress of both DNA polymerases [2, 3] and the RNA polymerase [4, 5]—and that therefore need to be removed for the cell to survive. Since UV is a part of the sunlight, all terrestrial organisms possess multiple repair systems that remove PDs and restore inhibited replication [6]. The two universal DNA repair systems addressing these problems are nucleotide–excision repair and recombinational repair. In bacteria, nucleotide–excision repair employs the UvrABC excinuclease that excises a short stretch of a PD-affected strand, so that the resulting excision gap can be filled in by using the intact strand as a template [7]. Again in bacteria, recombinational repair either fills in daughter-strand gaps behind replication forks, in a RecA-dependent process using the intact sister duplex as a template, or restarts PD-stalled replication forks via RecBCD-promoted linear DNA degradation, either to stabilize a regressed replication fork or to repair a disintegrated replication fork with the help of the same RecA protein [8]. Wild type (WT) *Escherichia coli* cells can survive 40 J/m^2^ of UV, which translates into ∼2400 PDs per genome-equivalent [9], but if both of these repair strategies are blocked, the resulting *uvrA recA* double mutant cannot survive a single PD in its chromosome [10].

For a long time, the only UV-sensitive mutants known in *E. coli* belonged either to PD removal or recombination-based pathways, with one notable exception of the *lexA* mutants [6]. LexA is a transcriptional repressor of the SOS response, which *E. coli* cells mount in case of massive DNA damage [11]; it initiates by the RecA filament catalyzing LexA self-cleavage [12, 13]. SOS-induction increases expression of ∼30 genes (among them *recA* and *uvrA*) and allows *E. coli* to boost DNA damage tolerance and DNA repair capacities [11]. Once the DNA damage is removed, normal expression patterns of the SOS genes are quickly restored, since the *lexA* gene is under self-regulation [14, 15] and is also highly expressed during SOS-induction [16].

We reported previously that RNase H-deficient mutants in *E. coli* (the *rnhA rnhB* doubles, or *rnhAB*, as we call them) turned out to be highly UV-sensitive (as sensitive as the *recBCD* mutants), yet are fully proficient in PD excision and DSB repair—which implied an unknown post-UV problem for chromosomal replication [17]. The *rnhAB* mutants cannot remove the two possible types of RNA/DNA hybrids (RDHs) [18] and therefore accumulate RDH signal in their chromosomal DNA (Supplementary Fig. S1A). All cells, whether eukaryotes or prokaryotes, have RNase H activities of two types, to keep DNA free of ribonucleotides [19, 20]. RNase HI (encoded by the *rnhA* gene in *E. coli*) hydrolyses RNA in RDHs of the long type, called R-loops—these are created during transcription when nascent transcripts hybridize with the template DNA strand, displacing the complementary DNA strand in a single-strand loop [21]. R-loops are stimulated by hyper-negative supercoiling, which accumulates in the DNA behind transcribing RNA polymerases [22]; conversely, they are destabilized by physiological levels of positive supercoiling [23]. Unremoved R-loops, detected as a modest increase in RDH signal in the *rnhA* mutants (Supplementary Fig. S1A) [17, 24], are apparently problematic, as the *rnhA* mutants show slower growth, replication initiation at other places of the chromosome besides *oriC* and even a slight UV-sensitivity [17, 25–27]. Because of these alternative initiations, *rnhA* mutants are able to (slowly) replicate even when their *oriC* is inactivated or deleted [26, 28].

RNase HII (encoded by the *rnhB* gene in *E. coli*) attacks the second type of RDHs, RNA nucleotides integrated into DNA strands, by incising 5′ at the rN/dN junction [29]. The physiological role of RNase HII in *E. coli* is unclear, since the *rnhB* mutants by themselves are not known for any defect, other than accumulation of infrequent singular rNs in their DNA [18]. However, they do have phenotypes in the *rnhA* mutant background: compared to the *rnhA* single mutants, the *rnhAB* double mutants have exacerbated replication problems, slow growth, high SOS induction, higher chromosomal fragmentation, perturbed nucleoid appearance, sensitivity to hydroxyurea and UV [17, 18, 30], suggesting that RNases HI and HII provide alternative pathways addressing the same chromosomal problems. We concluded before that *rnhAB* mutants are UV-sensitive due to replication–transcription conflicts, since their UV sensitivity was suppressed either by blocking transcription initiation with rifampicin or by the rpoB*35 point mutation [17], which destabilizes transcription elongation complexes [31, 32].

It is widely assumed that replication forks running into R-loops can lead to DSBs [33, 34]. Indeed, the *rnhAB* mutants in *E. coli* suffer from replication inhibition during exponential growth and are depended on recombinational repair (in particular, on RecA and RecBCD) for mending the resulting DSBs at replication forks [18]. However, we found no additional UV-induced DSBs in the irradiated *rnhAB recBC* (Ts) mutants, when compared to UV-irradiated *recBC* (Ts) mutants [17]. Instead, we observed that UV-induced RDH signal accumulation in the *rnhAB* mutants correlates with their inability to restore normal levels of post-UV replication—therefore, we proposed that RDHs block the progress of replication forks without causing their disintegration [17].

**Figure 1.**
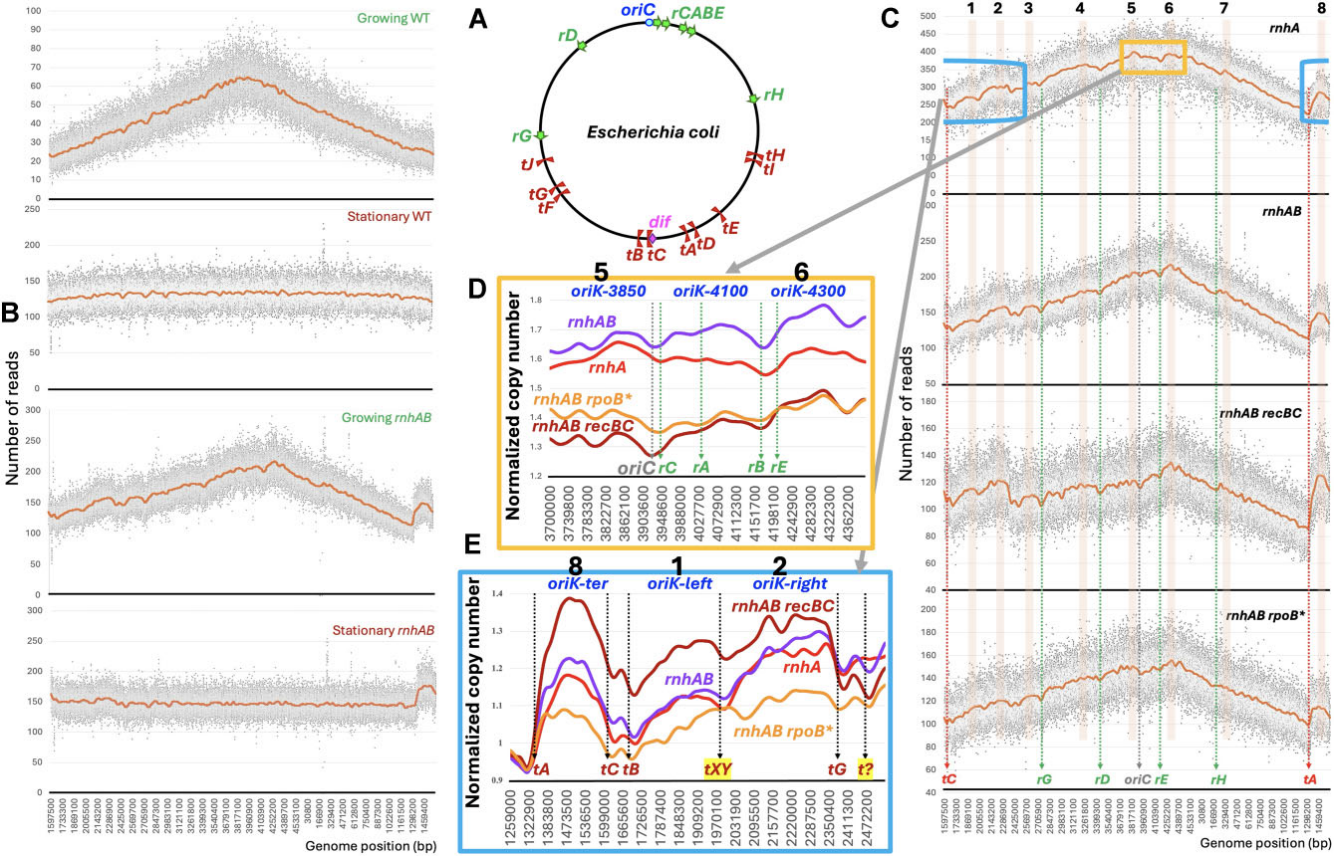
The complexity of replication profiles in cells lacking RNase H activities. Strains are: AB1157, L-413, L-416, L-476, and L-416–33. (**A**) A scheme of the *E. coli* chromosome, describing our “WT” AB1157, as well as the real WT, MG1655 (for the actual chromosomal coordinates, see Supplementary Table S1). The chromosome circle can be roughly subdivided into the upper half and the lower half. The upper half is centered on the replication origin, *oriC*, and is dominated by the ribosomal RNA operons, *rrnA-G* (green arrows marked *rA…rG*). The lower half is centered on the *dif* monomerization site in the middle of the terminus and is dominated by the ten replication termination sites (brown brackets marked *tA…tI*). (**B**) Comparison of the next-generation sequencing (NGS) replication profiles of WT and the *rnhAB* mutant strains, either growing at 28°C or stationary. To transition from the circular chromosome presentation in “A” to the linear one in replication profiles, the chromosome is opened at *dif* (see panel “A”) and presented as a line segment, with *oriC* positioned in the middle. As a result, *X*-axis is permuted for actual chromosome coordinates. This profile presentation, which we call “complete” includes both absolute read counts of 100 bp bins (dots) and LOESS regression curve (brown line, span = 0.025) which are plotted against genomic coordinates. (**C**) Profile comparison of the logarithmically growing mutants lacking RNase H activities, some with additional defects (all cultures grown at 28°C), to reveal the shared features. From top to bottom: *rnhA* single, *rnhAB* double (the same as in panel “B”), *rnhAB recBC*, rnhAB rpoB*. At the bottom, the shared profile troughs are marked by the associated loci, including *oriC*, the two major termination sites *terA* and *terC*, as well as the four evenly-spaced *rrn* operons, G, D, E, and H. At the top, numbers from 1 to 8 and the corresponding light brown columns mark the shared broad peaks, associated with *oriK* initiation zones. In the top profile, the yellow box delineates the origin macrodomain shown in detail in panel (D), while the light blue box (transposed because of the linear chromosome representation) delineates the terminus macrodomain detailed in panel (E). (**D**) Details of the four profiles in the origin macrodomain (chromosomal coordinates 3700–4400 kb). The *oriK* zones are labeled at the top in blue, while the features associated with troughs are marked at the bottom. LOESS values of all the profiles are normalized to the corresponding mean LOESS values in the *terE-terD* interval (see panel “A”). (**E**) The same as in “D,” but for the terminus macrodomain (coordinates 1259–2534 kb). More explanations in the text.

In the wild-type *E. coli*, chromosome replication initiates by the DnaA protein polymerization at a specific site called *oriC* [35, 36], from which two replication forks then move bidirectionally [37], meeting and fusing in the terminus region near the unidirectional termination site *terC* (Fig. 1A) [38, 39]. Replication from *oriC* is codirectional with highly transcribed genes, such as the seven ribosomal RNA (*rrn*) operons (Fig. 1A), to avoid deleterious head-on transcription-replication conflict [40–42]. Still, since, for example in rapidly growing *E. coli*, a typical rate of transcription is some 20 times slower than the maximal rate of replication [43] even codirectional replication–transcription conflicts are unavoidable— although barely detectable in WT cells ([44–47] and see Discussion). The situation dramatically worsens if an *rrn* operon is artificially inverted: the progress of replication forks across the head-on oriented *rrn* operons in bacteria is inhibited [27, 48, 49], while replication forks are destabilized [42, 50]. In fact, RNase H enzymes are required for replication forks to pass through a strong transcriptional unit in head-on orientation in *Bacillus* and in human cells [51, 52], highlighting a paradox of R-loop formation in the region dominated by positive supercoiling, expected to accumulate between converging polymerases [53].

As mentioned above, the *rnhA* mutants of *E. coli* can initiate replication throughout the chromosome without DnaA-protein—this is how they survive *oriC* deletion [28]. The initiation is catalyzed by PriA from accumulating R-loops and causes “constitutive stable DNA replication” (cSDR), characterized by its independence of translation—unlike the DnaA-dependent initiation at *oriC* [54]. Four prominent cSDR origins, called *oriK*s, were originally detected by copy number chromosome profiling [55]. The high-resolution marker-frequency-analysis in the *rnhA* mutants, blocked for *oriC* initiation, mapped additional *oriK*s [24, 26, 27]. Surprisingly, even with functional *oriC* in the *rnhA* mutant, the major replication peak was observed away from *oriC*, at the site downstream of the *rrnCABE* operon cluster (Fig. 1A), placing it in the head-on orientation to this powerful replication barrier—which somehow did not interfere with the overall chromosomal replication. Besides that, the most prominent *oriK* was always reported in the terminus, between the *terA* and *terC* sites (Fig. 1A) [24, 26, 27, 55]. Interestingly, attempts to clone *oriK*s by selecting for chromosomal regions able to drive plasmid replication in the *rnhA* mutants were unsuccessful [56, 57].

In this study we have characterized the post-UV replication dynamic patterns in the *rnhAB* double mutants with high-resolution chromosome marker frequency analysis, to identify specific chromosomal regions where forks could be stalling. Replication profiling of various *rnhA* mutants before UV revealed that *oriK*s are not specific initiation sites but rather represent broad initiation zones separated by occasional hard-to-replicate regions. Remarkably, in all *rnhA* mutants tested, post-UV initiation happened strictly from a single DnaA-dependent *oriK* ∼75 kb away from *oriC*. We also found that in UV-irradiated cells, RNase H activity becomes important for overcoming replication fork stalling at the *rrn* operons. In the absence of both RNase H enzymes and RecBCD helicase/nuclease, some *rrn* operons become impassable choke-points for replication forks, essentially blocking fork progress. In contrast to the reported before “physiological” replication–transcription conflicts only in the head-on orientation, the UV-induced replication–transcription conflict blocks forks in both orientations.

## Materials and methods

### Bacterial strains

*Escherichia coli* strains (all K-12) are described in Supplementary Table S3, while plasmids are described in Supplementary Table S4. Strain construction was by P1 transduction [58], Δ*rrnD, ΔrrnG, ΔrrnH* operons, and *ΔoriC* were constructed by Lambda-Red recombineering method with subsequent removal of the antibiotic resistance by pCP20 [59]. Deletions–replacements were confirmed by polymerase chain reaction (PCR). The *recBC*(Ts) and *ΔrnhA ΔrnhB* mutants were confirmed by their characteristic UV-sensitivities, the *dnaA* mutant was verified for no growth at 42°C, the rpoB35* ΔrnhA ΔrnhB strain was confirmed as being UV resistant, all *rrn* deletions were verified by Southern analysis using PCR-amplified *rrsA* and *rrlA* radioactive probes [60]. Within individual experiments, all important mutations were additionally confirmed by whole-genome sequencing. All the primers used for constructing deletions and verification of an indicated chromosomal loci, as well as the primers for PCR-amplified radioactive probes, are listed in Supplementary Table S5.

### Media and growth conditions

Cells were grown in LB broth [10 g tryptone, 5 g yeast extract, 5 g NaCl per liter, then pH 7.2 with NaOH] or on LB plates (15 g agar per liter of LB broth). The regular growth temperature was 28°C, unless otherwise indicated in the experiment description. When screening for mutations linked to antibiotic-resistant genes or when the cells were carrying plasmids, the media were supplemented with the required antibiotic: 100 μg/ml ampicillin, 50 μg/ml kanamycin, 10 μg/ml tetracycline,10 μg/ml chloramphenicol, and 30 μg /ml spectinomycin.

### UV irradiation

Two procedures used UV irradiation: UV survival and post-UV DNA analysis. For UV treatments, the time to deliver a particular UV dose was calculated from the direct measurements of UV irradiation fluency with UVC Digital Light Meter (model UV512C, GENERAL). All UV experiments were performed under yellow light (F15T8-GO lamp, General Electric) to avoid photoreactivation.

#### UV survival

Overnight cultures of the tested strains were diluted 100-fold in the morning and grown with shaking to OD_600_ ∼0.2 before UV irradiation. Ten-fold serial dilutions of the tested cultures were made in sterile 1% NaCl solution and spotted by 10 μl in one row for each dilution on several square Petri dishes with LB agar. Spots were dried, and each plate was exposed to a particular UV dose from a GE germicidal lamp emitting 254-nm UV light. Plates were developed overnight in the dark at 28°C, and colonies were counted under a stereomicroscope. The survival was determined as the ratio of a culture titer after a particular UV dose to the titer of the untreated culture.

#### Post-UV DNA analysis

Strains were grown in 20 ml of LB to OD_600_ of 0.4, collected by centrifugation and resuspended in 20 ml of 1% NaCl. Sixteen milliliters of cells were transferred to an open sterile glass tray (14 cm × 23 cm), placed under the GE germicidal lamp emitting 254-nm light and exposed to this UV light with a slow rotation of the tray on a platform. The only UV dose used in this paper was 40 J/m^2^. After irradiation, the cells were diluted (1:1) with 2 × LB without NaCl and transferred to a sterile flask for post-irradiation recovery at 37°C or 42°C (for the *dnaA46* strains), with vigorous shaking. “0 time” four milliliter aliquot was removed from the flask before placing the flasks at 37°C; other time points two or four milliliter samples were removed from flasks and placed on ice at specific times, then cells were collected by centrifugation and processed according to the “total genomic DNA isolation for deep sequencing,” “total genomic DNA isolation by sonication” or “Measurement of post-UV DNA replication by agarose plugs hybridization” protocols.

### Total genomic DNA isolation for deep sequencing

Cell lysates were prepared according to Brij lysis procedure [17]. Specifically, after described above UV exposure and post-irradiation recovery, cells were pelleted and resuspended in 0.25 ml of ice-cold solution of 30% sucrose, 0.05M Tris–HCl, pH 8.0. Freshly prepared lysozyme, 0.05 ml of 5 mg/ml in 0.25 M Tris–HCl, pH 8.0, was added to the cell suspension and kept on ice for 5 min with gentle mixing. 0.1 ml of 0.25 M EDTA, pH 8.0 was added for another 5 min on ice with occasional gentle swirling. To lyse the cells, 0.4 ml of detergent mixture (1% Brij 58, 0.4% sodium deoxycholate, 0.0625 M EDTA, and 0.05M Tris–HCl, pH 8.0) and RNase A to the final concentration 20 μg/ml were added and gently swirled. The samples were kept on ice for 30–60 min, followed by three organic extractions: 0.8 ml phenol with 40 μl chloroform, followed by 0.8 ml phenol/chloroform (1:1) mix, followed by 0.8 ml chloroform. The final aqueous phase was transferred into fresh tube and precipitated with salt and ethanol. DNA concentrations were measured with Qubit 2.0 Fluorometer (Invitrogen).

### Isolation of genomic DNA from agarose plugs

The cultures were grown and exposed to UV as described in “Post-UV DNA analysis” above. Cells from four ml culture (OD_600_ 0.2–0.5) were collected by centrifugation, resuspended in 100 μl of ice-cold TE buffer (10 mM Tris–HCl pH 8.0, 1 mM EDTA), then 20 μl of 5 mg/ml lysozyme was added. Cells were kept on ice for 5 min with gentle mixing, then 40 μl of 0.25 M EDTA pH 8.0 was added for another 5 min on ice with occasional gentle swirling. The tube was warmed at 37°C for 5 min, and cells were mixed with 160 μl of 1.2% low-melting-point agarose (SeaPlaque GTG agarose, FMC Bioproducts) in water. From this mixture, two plugs were cast in the plug mold, and the mold was kept on ice for 10 min for agarose to solidify. Out of the mold, each plug was placed in 1 ml lysis buffer (1% Brij 58, 0.4% sodium deoxycholate, 0.0625 M EDTA and 0.05 M Tris–HCl, pH 8.0) containing 20 μg/ml RNase A and incubated at 37°C for 30 min. Then, 50 μl of 5 mg/ml Proteinase K was added to the lysis buffer with the following incubation of the tube at 55°C O/N.

Next morning, the protocol from New England Biolabs for agarose digestion and DNA purification from agarose plugs was followed. Briefly, the agarose plugs were washed three times 30 min each in 1 ml of 10 mM Tris–HCl, pH 6.5, 1 mM EDTA on ice, then the buffer was similarly exchanged for 1× β-agarase I Buffer (twice) keeping tubes on ice for 30 min. The buffer was removed, and agarose plugs were melted at 65°C for 10 min and cooled to 42°C. β-Agarase I (NEB) was added to the molten agarose to a final concentration of 1 unit/100 μl following by incubation of the tube at 42°C for 2 h. After digestion, 5 M NaCl (1/10 of the volume) was added and incubated on ice for 30 min. Residual polysaccharides were precipitated by centrifugation at 15 000 *g* for 15 min. DNA-containing supernatant was transferred to a new tube, and DNA was precipitated with two volumes of isopropanol, followed by centrifugation. The DNA pellet was resuspended in 100 μl of TE buffer and extracted once with the same volume of chloroform, followed by precipitation with 0.5 M NaCl and two volumes of ethanol. Precipitated DNA was dried at room temperature, dissolved in water, and kept at –20°C.

### Total genomic DNA isolation by sonication and dot-blot analysis

The cultures were grown and exposed to UV as described in “Post-UV DNA analysis” above. Cells were collected from 5 ml of cell culture with OD_600_= 0.4 and resuspended in 50 μl of 30% sucrose in TE buffer. Three hundred and fifty microliters of 2% SDS in TE buffer was added; the tube contents were mixed by inversion and incubated at 70°C for 5 min to achieve a complete lysis. RNaseA solution was added to the cooled cell lysate to a final concentration of 20 μg/ml and the tube was incubated at 37°C for 30 min. Cooled cell lysates were sonicated in the “cup horn” attachment of Misonix S-4000 Sonicator Ultrasonic Liquid Processor using manual program with 50% power level and continuous pulse for a total of 6 min interrupted by a brief placement of tubes on ice every 2 min. After sonication 1.5 mg/ml (final concentration) of Proteinase K was added to the cell lysate and the tube was incubated overnight at 60°C. Next morning DNA was extracted by the standard phenol–phenol/chloroform–chloroform (1:1 ratio) procedure followed by DNA precipitation with 0.5 M NaCl and two volumes of ethanol. Precipitated DNA was dissolved in 100 μl of TE buffer. DNA concentrations were measured with Qubit 2.0 Fluorometer (Invitrogen). DNA samples were run on an agarose gel to confirm the breakage of chromosomal DNA to the 0.2–1 kbp in size. 0.5 μg of DNA was boiled in 100 μl of TE buffer for 5 min and split into two fractions. DNA was applied to the membranes (Hybond N+, GE), UV crosslinked to the membrane, and probed with radioactive probes for mdtP and uvrD regions. DNA images were generated with phosphor-imaging screens using Typhoon FLA 7000 (GE Healthcare) and calculated with ImageQuant TL program (GE HealthCare Life Sciences). The mdtP/uvrD signal ratio during post-UV recovery was calculated for each time point and normalized to the ratio obtained for the zero time (Supplementary Fig. S14B).

### Measurement of post-UV DNA replication by agarose plugs hybridization

To measure the absolute signal increase at specific chromosomal regions, we followed the previously published procedure [61]. Specifically, 1 ml aliquots of the culture treated with 40 J/m^2^ UV and shaking at 37°C were taken at 0, 60, 120, or 180 min post-UV. Cells were collected in Eppendorf tubes, spun down, and resuspended in 300 μl of TE buffer to make five agarose plugs. The procedure for making, treating, and hybridization of agarose plugs was as described [61]; ^32^P-labeled PCR-amplified fragments representing specific chromosomal regions were used for hybridization as described [62]. The signals from the plugs corresponding to 0, 60, 120, and 180 min were generated with Phosphorimager Typhoon FLA 7000 (GE Healthcare) and calculated with ImageQuant TL program (GE HealthCare Life Sciences). To calculate DNA increase after UV treatment, plug signals of the 60, 120, and 180 min were normalized to the signal of the plug at 0 min. The ^32^P-labeled *rrnG* probe (the “reporter” chromosome segment) is a combination of four PCR fragments with the total length of ∼44 kbp covering upstream and downstream sequences around the *rrnG* operon.

### Deep sequencing analysis

Library preparation and sequencing were performed by the Roy J. Carver Biotechnology Center at the University of Illinois Urbana-Champaign. Shotgun genomic libraries were prepared from purified genomic DNA with the Illumina DNA Prep kit (Illumina cat no. 20060060). Libraries were pooled, quantified by quantitative PCR (qPCR), and sequenced on one SP lane for 101 cycles from one end of the fragments on a NovaSeq 6000 with V1.5 sequencing kits.

Sequence data were trimmed and analyzed for quality with fastp (v0.23.4) [63] using default settings and aligned to the MG1655 genome (accession: NC_000913.3) using bwa-mem2 (v2.2.1) [64]. SAM files containing aligned reads were converted to BAM format and sorted to absolute genomic coordinates with Samtools (v0.1.17) [65]. Per-base read depth was derived from sorted BED files by the Genomecov module of the BEDTools software package (v2.31.0) [66].

Subsequent data manipulations were performed by custom scripts written in Python, R and Perl (available upon request). Per-base read depth was averaged over contiguous 100-base bins so that the dataset could be efficiently manipulated in Excel. Bins containing non-uniquely mapped reads were removed (1249 bins, including all *rrn* operons), and when the AB1157 (accession: NZ_CP076404.1) background was employed, an additional 624 bins overlapping AB1157-specific deletions were also removed. The binned and filtered dataset was linearized at the *dif* locus and padded for trendline generation by duplicating the terminal 1000 bins to reciprocal ends of the dataset. A LOESS trendline was then generated on the padded data by the “loess” function of the R Stats package using 8 fitting iterations with settings: degree = 2, family = symmetric, surface = direct. The value of “span” determines how responsive LOESS is to local variation, and it critically affects the trendline’s ability to properly capture signal while rejecting noise [67]. We sought an unbiased method to determine an optimal value for span, such that LOESS would best capture the signal in our data. To do so, we queried multiple representative datasets by the “gcv” (generalized cross validation) and “aicc” (Alkaline information criterion) bootstrap methods provided by the “loess.as” function of the fANCOVA R package [68], with options: degree = 2, family = symmetric, and otherwise default settings. Somewhat surprisingly, we found that span = 0.05, corresponding to ∼230 000 bp of genome sequence, was almost universally the best value for span and appeared optimal regardless of growth phase (stationary and exponential) or accumulated genome damage (UV dosage). However, this statistically optimal kernel window mostly obscured well established biological signals, such as fork stalling at *ter* sites and *rrn* operons. Therefore, a LOESS span value of 0.025 (115 000 bp) was used unless otherwise indicated. Binning the dataset either before or after LOESS generation did not significantly affect the generated trendline. After LOESS generation, the 1000-bin padding was trimmed, and the data exported to Excel for graphing.

To combine replication profiles for five independent UV-experiments (Supplementary Fig. S5), the binned data were normalized for sequencing depth by conversion of absolute read depth to percent mean read depth (the mean across all bins). LOESS trendlines were then generated on the normalized data, and the resulting trendlines averaged. Averaging of normalized datasets prior to LOESS generation produced virtually identical summary trendlines.

To build the nested sets, LOESS values were plotted against genomic position using the MG1655 reference genome. To position the four replication (marker frequency) profiles from a particular post-UV experiment in one replication profile chart, such that the height of each curve accurately reflected the relative loci-specific increases or decreases as determined by Southern analysis, we first normalized LOESS values of each sample to the LOESS mean value calculated from the *rrnG* region with coordinates 2700000–2748600. Second, we multiplied normalized LOESS values by the “absolute *rrnG* increase factor” calculated as described in *Measurement of post-UV DNA replication by agarose plugs hybridization*. In one particular comparison (Fig. 5D), LOESS values were normalized to the LOESS mean value calculated from *terE-terD* region with coordinates 1082000–1282000.

## Results

### The *oriK*s zones in the growing *rnhA* and *rnhAB* mutants are bracketed by either *ter* sites or *rrn* operons

In terms of the nature of potential replication-affecting features, the *E. coli* chromosome can be divided into two halves: the origin-centered and ribosomal RNA (*rrn*) operons-dominated top half versus the terminus-centered and termination (*ter*) sites-dominated bottom half (Fig. 1A and Supplementary Table S1). To facilitate presentation of replication profiles, coordinates of the linearized chromosome represent the *X*-axis, while the number of reads of the corresponding sequences is plotted on the *Y*-axis (Fig. 1B). To place the origin in the middle of the *X*-axis, we open the chromosome at the monomerization *dif*-site near *terC* (Fig. 1A).

In the growing WT *E. coli* cells, the replication origin is identified as the peak between the two symmetric and smooth slopes, with the minimum at *dif*, while stationary cells yield an essentially flat profile, indicating no replication activity in the aligned chromosome (Fig. 1B, the top two profiles). In the growing *rnhAB* mutant, the profiles are expected to be similar to the known *rnhA* single mutant profiles, which show significant differences from the WT profiles [26, 27]—and indeed they are (Fig. 1B, the two bottom profiles). Specifically, in the growing *rnhAB* double mutant cells, several broad “bumps” (the major one in the terminus) delineated by narrow troughs suggest additional replication–initiation zones separated by replication-inhibiting sites. Also, the dominant replication origin is shifted some 400 kb to the right of *oriC* (Fig. 1B), like in the *rnhA* single mutant [26, 27]. In the stationary *rnhAB* double mutant cells, even though the overall profile becomes flat, the terminus “bump” is still present (Fig. 1B), indicating an actual replication–initiation activity there leading to formation of a stable replication bubble [69].

As already mentioned, the *rnhA* single mutant profiles are known for these additional initiation zones, called *oriK*s. To identify common *oriK*s, we compared unchallenged replicating profiles of *rnhA*, *rnhAB*, *rnhAB recBC*, and rnhAB rpoB* mutants (Fig. 1C). This comparison identified eight major *oriK* zones, labeled 1–8 at the top (Fig. 1C), whose position correlates well with the published data (Supplementary Table S2). We also saw that most of the troughs defining *oriK* zones coincide with either *ter* sites or with *rrn* operons (even though all the bins with ribosomal DNA sequences were removed from the profile analysis as repeats). Interestingly, the four *rrn* operons, G, D, E, and H, are uniformly spaced and symmetrically arranged around the *oriK* zone #5, their position relative to the nearby *oriC* being somewhat asymmetric (Fig. 1A and C). Finally, *oriC* itself is in a trough in all the four strains, while the dominant peak is away from it, in the *oriK* zone #6 (Fig. 1C).

We inspected in more detail the two broad regions (Fig. 1C): around the origin (the yellow box covering the *oriK* zones 5 and 6) and around the terminus (the blue interrupted box covering the *oriK* zones 1, 2, and 8). In the origin region, *oriC* coincides with a conspicuous trough (Fig. 1D), as if, besides being inactive, it also interferes with fork progress (perhaps, due to *rrnC* nearby). Instead of *oriC*, the *rnhA* mutant profiles in the origin region are dominated by three initiation zones, which we labeled *oriK-3850*,*oriK-4100*, and *oriK-4300* (Fig. 1D). The reality of *oriK-4100* zone is confirmed by the averaged *rnhAB* profile (Supplementary Fig. S1B), making it a total of 9 *oriK* zones in the chromosome. Each *oriK* zone in the origin macrodomain comprises two separate bumps (Fig. 1D), so it does not represent a specific initiation site. In fact, the whole yellow box could have been a single continuous initiation zone, if not cut by two troughs, one at the *oriC-rrnC* region, the other at the *rrnBE* region (Fig. 1D).

In the WT cells, the terminus proper is the *terA-terC* region, which remains inconspicuous (Fig. 1B), unless the WT cells are recovering from DNA damage [70]. In the *rnhA* mutants, this region is known to be always overreplicated [24, 26, 27], yielding a characteristic bump (Fig. 1B and C), which we call *oriK-ter* (Fig. 1E). Our high-resolution analysis shows that, in the *rnhA* mutants, the terminus proper extends past *terC* to *terB* on the right (Fig. 1E). We also found that, adjacent to *oriK-ter*, there are two more broad initiation areas, which we call *oriK-left* and *oriK-right*, between *terB* and *terG* (Fig. 1E). Like in the origin macrodomain, these two areas could be a single one, but they are split by an unknown (termination) site in the middle (marked *tXY* in Fig. 1E). Interestingly, there are two *ter*-like sequences at this location, which we call *terX* and *terY*, differing by only two nucleotides from the *ter* consensus (Supplementary Fig. S1C) and in the opposite orientation to each other, so the pair could potentially inhibit fork progress in either direction. We tentatively call the location *terK* (Supplementary Fig. S1B), pending investigation into which of the two *ter*-like sites (or both?) is responsible for the local trough.

In conclusion, in the *rnhA* and *rnhAB* mutants, *oriC* seems inactive, while the growth replication profiles are “bumpy.” We identified 9 major *oriK* initiation zones, three of them in the origin macrodomain, but could not assign any specific features/genes/sites responsible for any of them. In contrast, the narrow troughs that delineate these broad *oriK* zones all coincide with either *ter* sites or *rrn* operons (Fig. 1).

### The WT and *rnhA* single mutant restore normal replication profiles in 3 h post-UV

**Figure 2.**
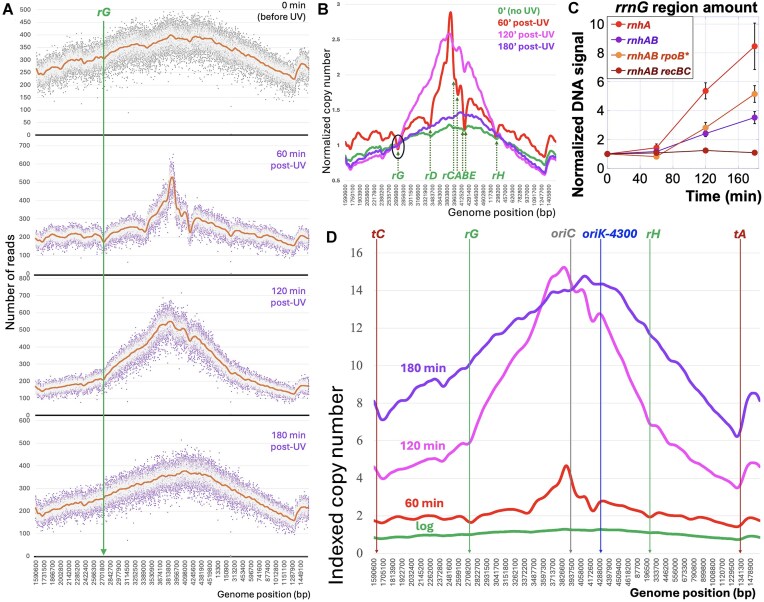
The post-UV replication recovery in the *rnhA* mutant. The strain: L-413. (**A**) The “complete” profiles of the *rnhA* mutant at 28°C (0 min), as well as during post-UV recovery at 37°C after 60, 120, and 180 min. A logarithmically growing culture was exposed to 40 J/m^2^ UV and then was continued shaking at 37°C. Genomic DNA was prepared from culture aliquots at the indicated time points for NGS. This will be the standard procedure and presentation throughout the paper. The *rrnG* operon position is marked; the region was used for normalization to convert reads to the gene copy number. (**B**) LOESS-only profiles from panel (A), normalized to the *rrnG* position (circled). Positions of all seven *rrn* operons are marked. (**C**) The restoration of post-UV replication in the four mutants of Fig. 1C. Increase in the absolute amounts of the “reporter” chromosomal DNA segment (44 kb on both side of *rrnG*) was measured at the indicated time points by plug-hybridization (see Materials and methods), to use for indexation of normalized profiles of the corresponding strains (like in panel B), to generate the nested set of profiles shown in panel (D). (**D**) The nested set for the *rnhA* mutant, based on panels (A–C). This will be another standard procedure/presentation throughout the paper. LOESS values (panel A), normalized to the LOESS averages from the *rrnG-*reporter region (panel B), were multiplied (“indexed”) by the actual increase in the DNA signal of the *rrnG* reporter for the *rnhA* mutant from panel (C).

We irradiate our cells with the standard dose of 40 J/m^2^ UV, which is still sublethal for WT cells [17], and collect replication profiles at 60, 120, and 180 min post-UV, building a set of indexed profiles (“the nested set”) by a procedure described for the *rnhA* single mutant below (Fig. 2). The pattern of post-UV replication recovery in WT cells (Supplementary Fig. S2) reveals a seeming overinitiation from the origin at 60 min post-UV, apparently to compensate for slow restart at existing replication forks. However, since the doubling time of WT cells in these growth conditions is <25 min, and the actual increase in the origin between 0 min (log) and 60 min post-UV is ∼5-fold, these numbers are consistent with normal initiation rate, peak sharpness being due to the slow progress of new forks. By 120 min post-UV, the chromosome replication is up to speed again in the origin half (Supplementary Fig. S2), and by 180 min the pre-UV shallow profile is restored, with the residual asynchrony of replication termination yielding the overreplication bump in the terminus (will be discussed elsewhere).

Since the *rnhA* single mutant is modestly sensitive to UV [17], we expected its post-UV profiles to reveal problems in replicating through certain chromosomal regions. Indeed, the raw profiles of the *rnhA* single mutant looked quite uneven at 60 min post-UV, with replication inhibition at *rrnG* and *rrnH* still visible even at 120 min post-UV—although by 180 min post-UV, the profile essentially returned to its pre-UV shape (Fig. 2A). Interestingly, in contrast to the broad initiation zone before UV, post-UV initiations were mostly from the *oriC* area seen as a narrow sharp peak until the profile returned to normal at 180 min post-UV, where the initiation zone broadened again (Fig. 2A). In our experience, when interpreting time series of the chromosome profiles, it is important to normalize them by the locus that changes the least [61, 71, 72]. Since the area around *rrnG* appeared to replicate minimally, we initially used it to normalize the four profiles, and the resulting patterns did provide some useful information, like minimal replication at all seven *rrn* operons (Fig. 2B). At the same time, we realized that the relationship between the four profiles in Fig. 2B could not be the correct one. For example, *rrnG* and *rrnH* regions appeared as replication choke-points—but this contradicted restoration of normal replication by 180 min (Fig. 2A).

Measurement of the absolute amounts of the “reporter” chromosomal DNA segment (22 kb upstream and 22 kb downstream of *rrnG*) at different time points post-UV showed its considerable increase in the *rnhA* single mutant (Fig. 2C). Using these values for indexation of the normalized profiles of Fig. 2B produced a readily interpretable nested profile set visualizing the dynamics of post-UV replication of the entire chromosome (Fig. 2D). Such nested sets can be presented with either linear *Y*-axis (Fig. 2D), or with log *Y*-axis (Supplementary Fig. S3A). The plus of the linear scale is that it shows the real numbers of the replication increase for any locus. The minus is that the multiplication of indexation distorts the relative shape of the profiles (Fig. 2D). The plus of the log scale is that it preserves the relative shape of the profiles, for example revealing the shape similarity between the “log” and 180 min profiles, indicating restoration of normal replication pattern (Supplementary Figs S2 and S3A). The minus is that it makes the relative numbers harder to read.

In conclusion, at 60 min post-UV, the replication profile of the *rnhA* single mutant becomes bumpy due to the increased activity of pre-existing *oriK*s (Fig. 2A). Interestingly, a closer examination reveals that the dominant post-UV initiation peak is not at *oriC*, but is slightly shifted to the left, moving into the *oriK-3850* zone (cf. Figs 1D and 2D). However, in 180 min post-UV, there is a complete restoration of normal replication profile, with the dominant initiation shifting back to *oriK-4100* and *oriK-4300* (Fig. 2D). We also conclude that indexation of the normalized profiles yields readily interpretable time sets, which we therefore employ through the rest of this paper.

### The *rrn* operons impede replication in the *rnhAB* double mutant after UV

**Figure 3.**
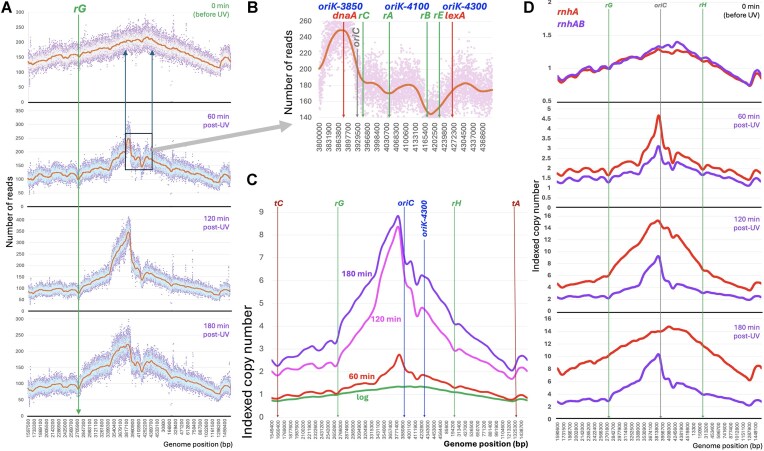
Impeded post-UV replication recovery in the *rnhAB* mutant. The strain: L-416. (**A**) The complete profiles of the *rnhAB* mutant at 28°C (0 min), as well as during post-UV recovery at 37°C after 60, 120, and 180 min. (**B**) The replication patterns at 60 min post-UV in the 3800–4400 kb segment of the chromosome around *oriC-oriK-4300* (boxed in “A”). (**C**) Nested profile set for the *rnhAB* mutant, based on panel (A) and generated as in Fig. 2D. (**D**) Comparison of post-UV profiles of the *rnhA* single mutant (strain L-413) versus *rnhAB* double mutant. The comparison is done by time points (0–60 to 120–180 min) using the indexed copy number profiles from Fig. 2D versus Fig. 3C.

The main goal of this project was to profile post-UV replication in the *rnhAB* double mutant and to identify possible harder-to replicate chromosome regions. Not much action is observed up to 30 min post-UV in the double mutant (Supplementary Fig. S4A–C), but by 60 min post-UV, the mutant begins to replicate its chromosome. The 60 min post-UV profile in this mutant is as uneven as the profile of the *rnhA* single mutant (cf. Figs 2A and 3A), but, in contrast to the later smooth profiles of the single mutant, the 120 and 180 min post-UV profiles in the double mutant stay uneven, showing little improvement. Like the *rnhA* single mutant, the *rnhAB* double also switches initiation from *oriK-4300* to what looks like *oriC* (Fig. 3A), but magnification of the origin region reveals that the UV-induced origin is in fact *oriK-3850* (Fig. 3B). Moreover, since the double mutant fails to restore normal replication, *oriK-3850* remains the dominant origin throughout its post-UV time course (Fig. 3A and C).

Looking at the complete profiles (Fig. 3A) we thought that the *rrnG* region does not replicate much after UV in the double mutant—therefore, we tried to use its normalization to build the profile set for comparison and interpretation (Supplementary Fig. S4D). The resulting pattern suggested a dramatic fork stalling at the *rrn* operons. However, the actual four-fold increase of the *rrnG* “reporter segment” after UV (Fig. 2C) indicated significant replication, prescribing indexation. The resulting nested set of the *rnhAB* mutant profiles shows robust initiation at *oriK-3850* combined with a limited replication progress along the chromosome (Fig. 3C). In fact, in the post-UV *rnhAB* mutant, the origin-proximal regions outreplicate the terminus-proximal regions 3- to 4-fold (Fig. 3C). The individual post-UV profiles of the *rnhAB* double mutant stay uneven, because all the troughs, originally present in the growing profiles of this mutant, are exacerbated after UV. In particular, the replication in the origin half of the chromosome (Fig. 1A) is severely impeded by the *rrn* operons, especially by the *rrnG* in the left replichore and by the *rrnCABE* cluster in the right replichore (Fig. 3B and C).

It should be noted that the post-UV replication profiles of the *rnhAB* mutant show good reproducibility (Supplementary Fig. S5). To address the possibility that the observed discontinuities at the *rrn* operons are artefacts of our standard DNA isolation protocol, we isolated genomic DNA from cells embedded in agarose plugs, the procedure that was shown to be less prone to such artefacts [73]. We observed no major changes between the two sets of profiles (Supplementary Fig. S4D), suggesting that discontinuities are not artefacts of differential DNA isolation.

Comparison of the nested sets of the single *rnhA* mutant with the double *rnhAB* mutant (Supplementary Fig. S3) suggests that the troughs originally present at 60 min post-UV in both mutants, all remain 180 min post-UV in the double mutant, while showing some interference with restoration of normal replication in the single mutant. To examine this idea, we built a time-point-specific comparison between the indexed *rnhA* and *rnhAB* profiles (Fig. 3D). While there is little difference in 0 and 60 min profiles between the two mutants, 120 min and especially 180 min profiles clearly show the inhibition of fork progress at the *rrn* operons in the double mutant. In particular, the block from the *rrnCABE* cluster makes post-UV replication at 120 and 180 min essentially a unidirectional one, similar to our prior experience with unidirectional origins [62]. We conclude that (i) the ribosomal RNA operons are the major inhibition-points for post-UV replication in the absence of RNase HI enzyme; (ii) the activity of RNase HII is necessary to eventually relieve this inhibition in the single *rnhA* mutant; (iii) in the absence of both RNase H enzymes in *E. coli*, the post-UV replication inhibition at the *rrn* operons becomes long-lived and severe.

### In the *rnhAB recBC* mutant, *rrn* operons block post-UV replication

And, yet, the nested set (Fig. 3C) clearly shows that the *rnhAB* double mutant is capable of significant, though incomplete, replication. What could be the main supporting factor for this (limited) replication progress? Since inhibited replication forks are unstable (known to break) [74, 75], the main supporting activity is expected to be recombinational repair of disintegrated replication forks [8, 76]. To test this idea, we introduced the *recBC* defect, blocking repair or degradation of linear DNA, into the *rnhAB* double mutant. Since unconditional *rnhAB recBC* mutants are synthetically lethal [18, 30], we used the *recBC* (Ts) allele and propagated the quadruple mutant at the permissive 28°C temperature. At the non-permissive temperature of 37°C, the quadruple mutant is static for several hours; specifically, our 180 min incubation at 37°C does not reduce its titer.

**Figure 4.**
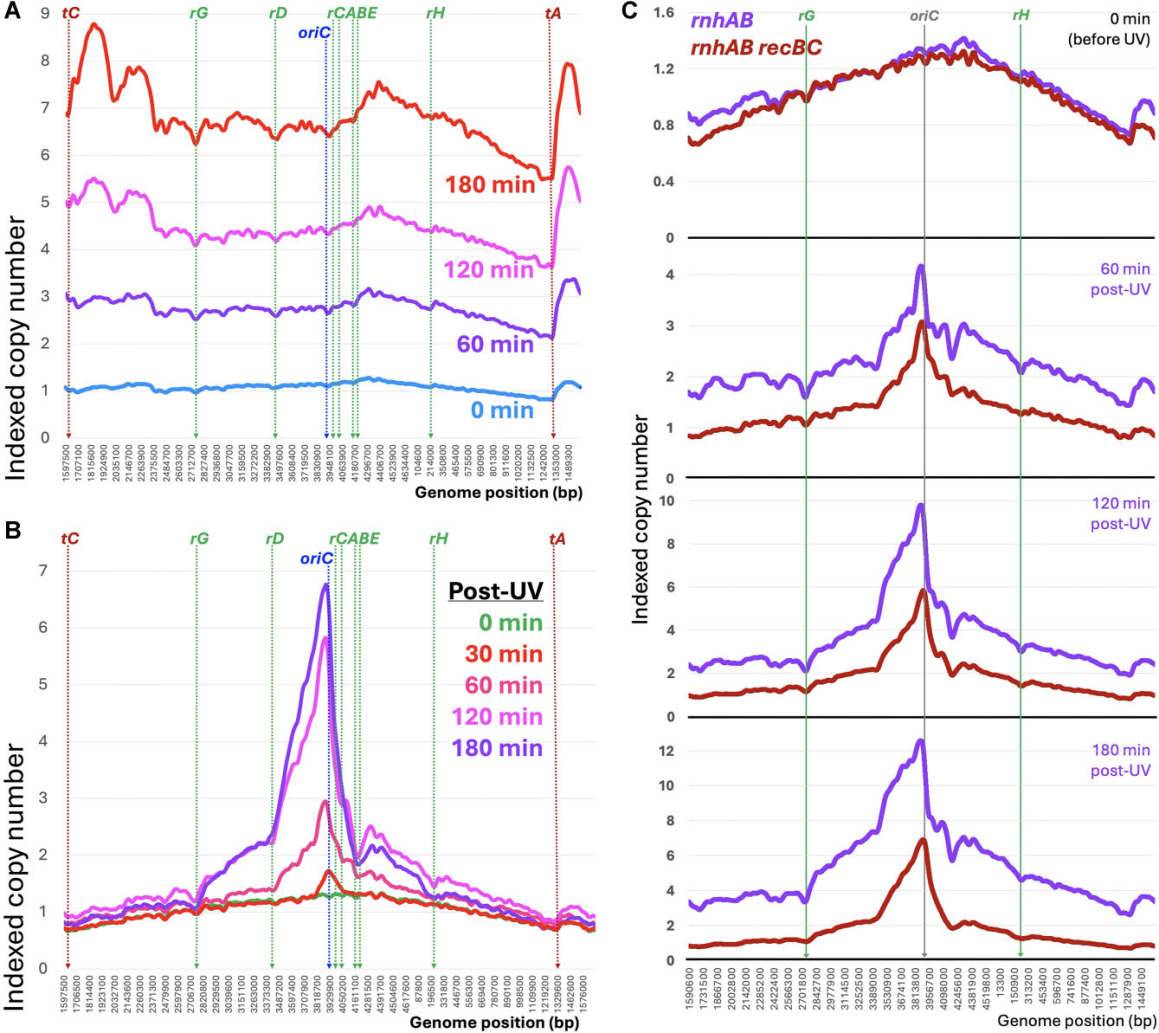
The nested sets of the *rnhAB recBC* mutant. The strain: L-476. (**A**) Replication of the *rnhAB recBC* (Ts) mutant *without* UV at its nonpermissive temperature of 37°C. A culture grown at 28°C was transferred to 37°C, and genomic DNA was prepared at the indicated time points for NGS. For the complete profiles, see Supplementary Fig. S6. (**B**) Replication of the *rnhAB recBC* (Ts) mutant after UV at 37°C, based on the complete profiles (Supplementary Fig. S7) and generated as in Fig. 2D. Both profiles in panel (A) and (B) are *Y*-linear. For their *Y*-log versions, see Supplementary Fig. S8. (**C**) Comparison of post-UV chromosomal replication of the *rnhAB* (strain L-416) versus *rnhAB recBC* quadruple mutants by time points using the indexed copy number profiles (Fig. 3C versus Fig. 4B).

Still, we first tested how the replication profile of the *rnhAB recBC*(Ts) mutant changes at 37°C without UV (Fig. 4A and Supplementary Fig. S6). It turns out that the mutant gradually slows down replication outside the terminus, especially in the left replichore, which is becoming quite uneven, and at the same time enhances replication within the extended terminus (Fig. 4A). Basically, initiation from the *oriK*s in the origin macrodomain is subsiding at 37°C, while initiation from the *oriK*s in the terminus half of the chromosome continues and, perhaps, is even enhanced.

The post-UV replication pattern in the *rnhAB recBC*(Ts) mutant, even though at the same temperature of 37°C, was dramatically different (Fig. 4B). In contrast to our expectations, based on Fig. 4A, the complete post-UV profiles looked similar to the *rnhAB* ones (cf. Supplementary Fig. S7 with Fig. 3A), with the sharp initiation peak in the origin region starting again at *oriK-3850* and a limited fork progress, in particular through the *rrnCABE* region. Quite startling was the complete lack of additional initiations in the terminus half of the chromosome—especially so since after this dose of UV, pyrimidine dimers are all removed by the WT strain in 60 min [17]. But there was a big difference from the *rnhAB* double mutant, too: the post-UV nested set of the *rnhAB recBC*(Ts) mutant revealed an extremely limited replication progress past *rrnG* in the left replichore and past *rrnH* in the right replichore, essentially turning these two origin-distal *rrn* operons into the post-UV replication choke-points (Fig. 4B).

The time-point comparison between the indexed *rnhAB* and *rnhAB recBC* profiles helps visualize the facilitating effect of double-strand break repair on the post-UV replication in the *rnhAB* mutant (Fig. 4C). The overall shape of the *rnhAB recBC* profiles faithfully repeats the general shape of the *rnhAB* profiles (Fig. 4C), but it is significantly and uniformly depressed compared to the latter, suggesting that post-UV initiation from *oriK*s all over the chromosome depends on RecBC (D). We conclude that the limited post-UV replication in the *rnhAB* mutant is mostly supported by repair or degradation of linear DNA, that is known to form at inhibited replication forks [75, 76]. At the same time, how UV manages to “flip” the replication profile of the entire chromosome, inducing initiations from an otherwise silent at 37°C *oriK-3850*, while all but suppressing robust initiations from the terminus half (Fig. 4A versus Fig. 4B and Supplementary Fig. S8), remains a mystery.

### UV-induced replication–transcription conflicts at the *rrn* operons

If the ribosomal RNA operons impede the progress of replication forks after UV, this is likely due to the replication–transcription conflict there, as transcribing RNA polymerase is one of the most stable protein–DNA complexes known [77]. Interestingly, the rpoB* mutants in *E. coli* that destabilize transcription elongation complexes were found to relieve UV-sensitivity of certain DNA repair mutants [31, 32]. We also observed that, in contrast to its *rnhAB* parent, the rnhAB rpoB* mutant is UV-resistant, confirming the critical role of transcription in making the *rnhAB* mutant UV-sensitive [17]. Since, *in vitro*, transcription complexes stalled at PDs protect them from NER [5], while, in the cells, the rpoB* defect allows the *rnhAB* mutants remove PDs with WT kinetics [17], the rpoB* destabilization likely works by making all PDs accessible to NER—as was also suggested before [32].

**Figure 5.**
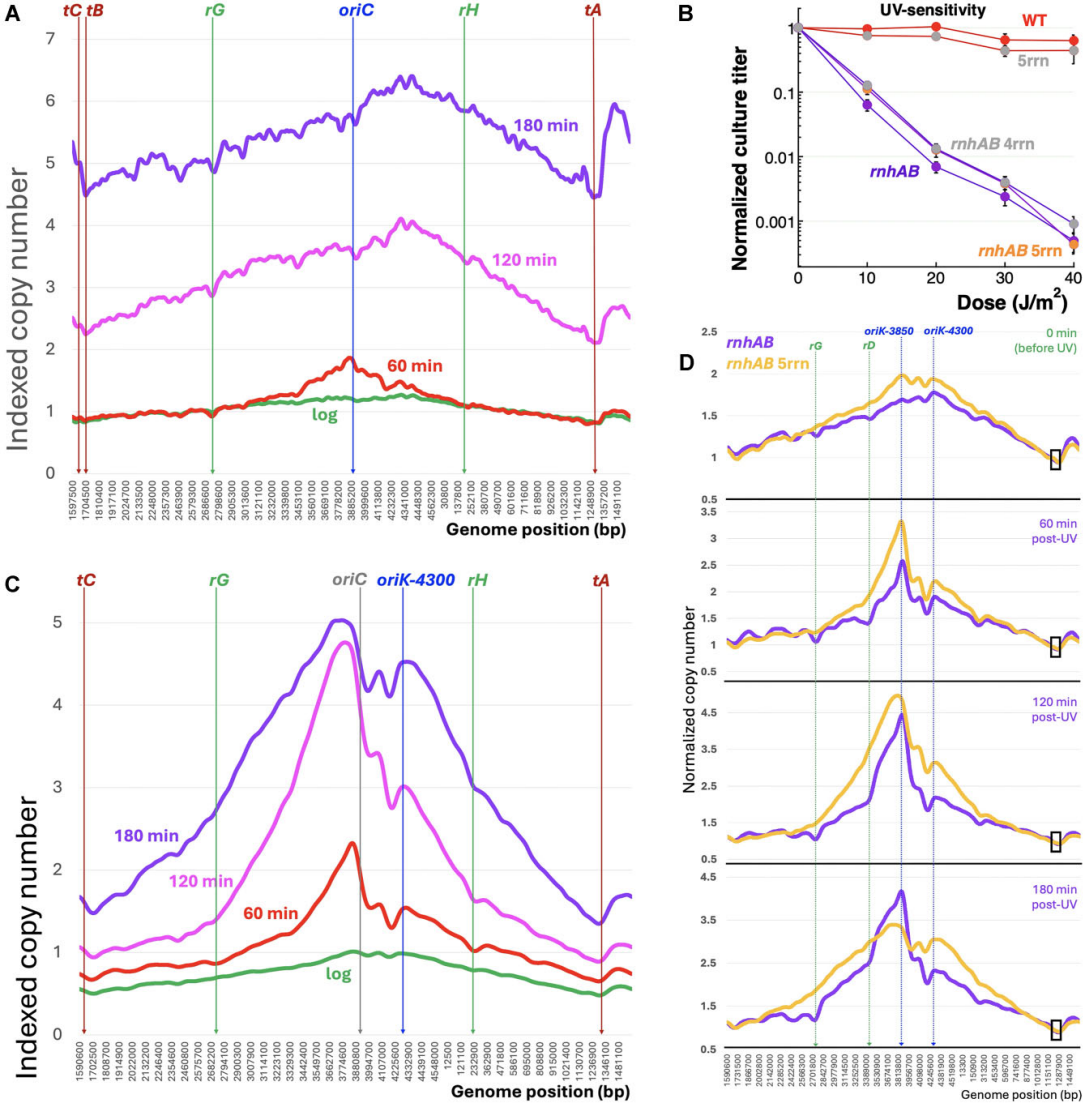
Post-UV replication restoration in the rnhAB rpoB* and *rnhAB* 5rrn mutants. (**A**) Nested profile set for the rnhAB rpoB* mutant after UV, based on Supplementary Fig. S9 and generated as in Fig. 2D. The strain: L-416-33. (**B**) UV-sensitivity of the 4rrn (Δ*rrnG* Δ*rrnD ΔrrnH*) and 5rrn (Δ*rrnG* Δ*rrnD*) strains in the WT and the *rnhAB* backgrounds. Strains: WT, AB1157; 5 rrn, L-531–2; *rnhAB*, L-416; *rnhAB* 5rrn, L-534–2; *rnhAB* 4rrn, L-554. (**C**) Nested replication profile set for the *rnhAB* 5rrn mutant after UV, based on Supplementary Fig. S12 and generated as in Fig. 2D. (**D**) The comparison of the post-UV replication profiles of the *rnhAB* mutant and its 5rrn variant (Δ*rrnG* Δ*rrnD*). The LOESS values in each pair are normalized by the corresponding mean LOESS value from the *terDE* region (boxed). The positions of the two deleted *rrn* operons are marked. The strains: L-416 and L-534–2.

The unchallenged replication profile of the rnhAB rpoB* mutant is not much different from the profile of its *rnhAB* parent (Fig. 1C). Even the 60-min post-UV profile of this mutant (Supplementary Fig. S9) also looks similar to the analogous profile of the *rnhAB* mutant (cf. Fig. 3C versus Fig. 5A or Fig. 3A versus Supplementary Fig. S9D), featuring robust initiation from *oriK-3850*. However, already the 120 min post-UV profile of rnhAB rpoB* starts resembling its normal replication profile, due to the initiation switch from *oriK-3850* back to *oriK-4300* (Supplementary Fig. S9). In fact, the nested set of the rnhAB rpoB* mutant shows that the mutant does return to normal replication already at 120 min post-UV (Fig. 5A), which is even faster than the WT replication recovery by 180 min (Supplementary Fig. S2). We conclude that the problems with post-UV replication recovery in the RNase H-deficient background are indeed due to the replication–transcription conflicts.

If the ribosomal operon transcription after UV can indeed block fork progress in the whole replichore, then deletion of individual ribosomal operons should significantly unblock this progress and may even ease the UV-sensitivity of the *rnhAB* mutant. Making use of the fact that deletions of up to three *rrn* operons do not affect growth of *E. coli* [78, 79] and that the whole left replichore has only two operons, *rrnG* and *rrnD* (Fig. 1A), we deleted them in our *rnhAB* mutant, yielding its *rnhAB* 5rrn derivative. We also deleted *rrnH* from the 5rrn strain to yield the *rnhAB* 4rrn mutant. All deletions were verified by Southern analysis (Supplementary Fig. S10) and genomic sequencing. As expected, deletions of the two *rrn* operons in the RnhA + RnhB+ (WT) strain did not affect its replication profile (Supplementary Fig. S11). But against our expectations, deletion of two and even three *rrn* operons failed to improve the UV-resistance of the resulting *rnhAB* mutant strains (Fig. 5B).

The growth profile of *rnhAB* 5rrn strain initiates from all three *oriK*s of the origin macrodomain (cf. Supplementary Fig. S12 top panel with Fig. 1D), while the post-UV profiles indeed show a smooth left replichore (Supplementary Fig. S12). In fact, the nested profile of the strain (Fig. 5C) not only features an improved and trough-less left replichore but, surprisingly, also shows less replication impediment in the right replichore, at the *rrnCABE* and *rrnH* operons. Both improvements are especially clear in comparison with the *rnhAB* mutant profiles, paired by post-UV times (Fig. 5D). The improved post-UV replication in the right replichore by deleting the two *rrn* operons in the left replichore supports the formation of the “bacterial nucleolus,” where all seven rrn operons come together for expression and processing of the nascent rRNA [80, 81]. In other words, reducing the strength of the “nucleolus” by reducing the number of participating *rrn* operons reduces the overall impediment to replication through all *rrn* chromosomal regions. At the same time, the overall post-UV replication progress remains as limited in the 5rrn construct as in its *rnhAB* parent, and, surprisingly, even with the *rrnG* and *rrnD* troughs gone, the left replichore progress remains fairly symmetrical with the right replichore (Fig. 5D), even though the latter is still blocked by the remaining five *rrn* operons. We conclude that, even though *rrn* regions are significant barriers for replication forks after UV, transcription of the rest of the chromosome still provides enough impediment to kill the *rnhAB* mutant, and this cannot be relieved by deleting individual *rrn* operons.

### Does *oriC* play а role in the post-UV replication initiation?

**Figure 6.**
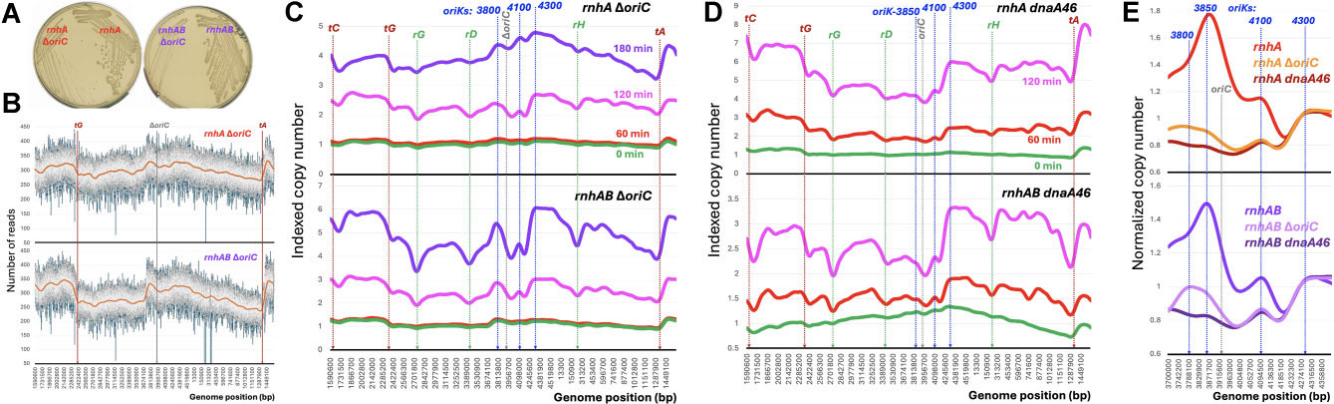
Effect of the *ΔoriC* and *dnaA* mutations on post-UV replication in the *rnhA* and *rnhAB* mutants. (**A**) Growth of *rnhA* (L-413), *rnhAB* (L-416), *rnhA ΔoriC* (L-555), and *rnhAB ΔoriC* (L-556) mutants, on LB plates for 2 days at 28°C. (**B**) The replication profiles during logarithmic growth of the *rnhA* Δ*oriC* and *rnhAB* Δ*oriC* mutants. (**C**) Nested sets of the post-UV replication profiles of the two Δ*oriC* mutants. (**D**) Nested sets of the post-UV replication profiles of the *rnhA dnaA*(Ts) (L-483) and *rnhABdnaA*(Ts) (L-484) mutants. (**E**) The UV response of *oriK-3850* relative to *oriK-4300* in the three variants of either *rnhA* single mutant (top) or *rnhAB* double mutant (bottom). The comparison is for 60 min post-UV recovery only. The three variants in both cases are *oriC*+ *dnaA*+, Δ*oriC dnaA*+, and *oriC*+ *dnaA46* (Ts). To enable comparison, the LOESS values in the origin macrodomain were normalized to the corresponding LOESS mean value of 50 kbp region at 4300 kb position. Strains: L-413, L-555, L-483 and L-416, L-554, L-484.

Since the origin of replication of the WT *E. coli*, *oriC* at position 3926 kb, seems non-functional in both the *rnhA* and *rnhAB* mutants, and *oriC* is known to be non-essential in the *rnhA* mutants [28], we decided to remove it, expecting that *oriK-3850*, the UV-induced origin in our *rnhA* mutants, would still fire in the absence of *oriC*. To this end, we deleted 2.1 kb region containing the minimal 232 bp *oriC* proper, as well as the surrounding *mioC*, *mnmG* (*gidA*), and *asnC* genes. Deletion of *oriC* in both *rnhA* and *rnhAB* mutants made the resulting strains grow really slowly (Fig. 6A). Since inactivations of the above *oriC*-surrounding genes are not known to affect growth, we attribute the slow growth phenotype to the inability of the DnaA replication–initiator protein to bind this region. The Δ*oriC rnhA* mutants were reported to grow slowly in rich media [28].

The profiles of growing cultures of the Δ*oriC* mutants looked flatter than those of their *oriC*+ parents, but *oriK-3850* (the small peak just to the left of *oriC*) was still functional (Fig. 6B and cf. Fig. 1C). Interestingly, after UV, *oriK-3850* was still modestly induced, but the dominant post-UV initiation in both mutants remained at *oriK-4300* (Fig. 6C)—meaning that the strong post-UV induction of *oriK-3850* in the *rnhA* mutants depends on *oriC* some 75 kb away. The time-point-specific comparison of *rnhA* and *rnhA* Δ*oriC* strains emphasizes the critical importance of the deleted 2 kb region containing *oriC* for the overall replication of the chromosome of the *rnhA* mutant, even in the terminus region (Supplementary Fig. S13).

The surprising *oriC*-dependence of *oriK-3850* suggested that initiation at the latter is achieved with the help of the DnaA initiator protein that polymerizes at *oriC*. We tested this possibility in the *rnhA dnaA*(Ts) and *rnhAB dnaA*(Ts) mutants at 42°C (the non-permissive temperature for our *dnaA46* (Ts) allele). The 28°C (permissive temperature) profiles of these mutants look similar to the unchallenged profiles of their DnaA + parents (Fig. 6D). The after-UV profiles of the *rnhA dnaA*(Ts) and *rnhAB dnaA*(Ts) mutants at 42°C remain essentially flat, although extremely uneven, with the terminus half replicating as much as the origin half. Importantly, there is no sign of *oriK-3850* post-UV induction, showing its complete DnaA-dependence (Fig. 6D).

Thus, instead of UV-induction of *oriK-3850* in the *rnhAB* (DnaA+) mutants (Fig. 3C), UV triggers formation of an *oriC*-centered wide lacuna in the profile of *rnhAB dnaA*(Ts) mutant at 42°C. The copy number difference between *oriC* and *oriK-4300* positions in this profile is so unexpectedly dramatic (Fig. 6D, the bottom profile) that we suspected this lacuna to be an artefact of incomplete DNA isolation of the *oriC*-centered chromosome region by our standard protocol (Supplementary Fig. S14A). To test the possibility of DNA under-isolation, we employed a pair of more stringent genomic DNA isolation protocols, measuring the ratio of two loci, one at position 4300 kb (*mdtP*, the high point), the other at position 4000 kb (*uvrD*, the low point) (Supplementary Fig. S14A). However, the more robust DNA isolation protocols confirmed the 1.5-fold difference observed at 120 min post-UV replication profile of the *rnhAB dnaA*(Ts) mutant (Supplementary Fig. S14B). Therefore, we conclude that there is no under-isolation of certain chromosome regions after UV, and the *oriC*-centered lacuna, formed due to *oriK-3850* inactivity, is real.

To explore these surprising findings further for both the *rnhA* mutant and the *rnhAB* mutant, we plotted origin macrodomain profiles at 60 min post-UV recovery of the three strains: *oriC + dnaA+*,*ΔoriC*, and *dnaA*(Ts), normalizing all three by their robust *oriK-4300* initiation zones (Fig. 6E). This normalization emphasized the differences between the Δ*oriC* and *dnaA* results: *oriK-3850* was still (barely) functional in the absence of *oriC*, but it completely fails to fire after UV without DnaA—suggesting the presence of DnaA boxes in the *oriK-3850* region (see Discussion).

## Discussion

We reported before that, while single *rnhA* mutants of *E. coli* show modest UV-sensitivity, the *rnhAB* double mutants are surprisingly UV-sensitive. Yet, they do not experience UV-induced *rnhAB*-dependent double strand breaks—instead, they have residual level of unremoved PDs—and fail to regain pre-UV rates of DNA synthesis [17]. In this work, we used chromosome profiling of the *rnhAB* double mutants to reveal possible problematic regions for their post-UV replication. Since regular replication in the *rnhA* single mutants is known to initiate from multiple *oriK*s [24, 26, 27, 55], we first mapped dominant *oriK*s during the unchallenged replication of the *rnhA* and *rnhAB* mutants. We found that, instead of *oriC* (position 3926 kb), three *oriK*s operate in the origin macrodomain, which we labeled by their approximate coordinates *oriK−3850*, *oriK−4100*, and *oriK−4300*. While *oriK-4300* tends to dominate during normal growth, initiation of post-UV replication in all *rnhA* mutants switches to *oriK-3850*. When the *rnhA* single mutant restores regular replication after UV, its initiation returns to *oriK-4300*. In contrast, the *rnhAB* double mutant struggles with post-UV replication fork progress, especially over the ribosomal RNA operons. In the *rnhAB recBC* mutant, that in addition cannot repair or degrade linear DNA, the post-UV fork progress slows down even further, especially over the ribosomal RNA operons, with the origin-distal *rrnG* and *rrnH* becoming chromosomal replication choke-points. On the opposite side of this spectrum, in the rhnAB rpoB* mutant that has unstable transcription complexes, the post-UV replication recovers even faster than in WT, confirming the replication–transcription conflict as the culprit. We also show that deleting the two *rrn* operons from the left replichore removes the corresponding slow-down points from the profile, but also, surprisingly, improves replication in the right replichore that still retains its five *rrn*s, indirectly confirming that *rrn* operons in *E. coli* cells form a single complex, called the bacterial nucleolus [80–82]. Although *oriC* appears to play no role in replication initiation in the *rnhA* mutants, its deletion dramatically reduces the overall replication activity in the chromosome and dampens the post-UV induction of *oriK-3850*. The remarkable post-UV initiation from *oriK-3850* is completely eliminated in the *dnaA* (Ts) mutants at 42°C, suggesting that this *oriK* depends not only on *oriC*, but also on DnaA. Below we discuss the general nature of *oriK* initiation zones, the UV-inducible *oriK-3850*, as well as possible mechanisms of UV-induced replication–transcription conflict.

### UV induces codirectional replication–transcription conflict

Our work with the *rnhAB* mutants argues for a new type of UV-induced DNA lesion (or rather a chromosomal scenario/situation), in which transcription plays a critical role. Previously, we reported [17] that our WT progenitor strain has <10% of the original PDs remaining at 30 min post-UV and no PD detected at 60 min post-UV. In contrast, the *rnhAB* mutants had slower and incomplete PD removal, so that ∼5% of the original PDs are still detected in the chromosome by 2 h post-UV [17]. At the same time, the stable RDH signal (detected with S9.6 antibodies) in the *rnhAB* double mutant was: (i) amplified by the restart of post-UV DNA synthesis at 60 min; (ii) enriched with PDs [17]. These observations allowed us to propose before that transcribing RNA polymerases, stalled at UV-induced PDs in template DNA, represent stable R-loop-anchored transcription–elongation complexes (R-loop-aTECs) [18, 53], which are further strengthened by the arrival of head-on replication forks. We further proposed that the R-loop-aTECs are impassable for head-on replication forks without RNase HI or RNase HII intervention and become the sites of severe post-UV replication–transcription conflicts. Since only head-on replication–transcription conflicts were known to be deleterious (see the Introduction) and since *rnhAB* mutants initiate replication at multiple *oriK*s around the chromosome (Fig. 1), we speculated that head-on replication–transcription conflicts could happen between *rrn* operons and forks from *oriK*s moving in the *oriC* direction [17]. The troughs at all seven *rrn* operons in the replication profiles of growing *rnhAB* mutants (Fig. 1) are consistent with this logic.

Further confirmation came from the observation that all *rrn* operons develop even more prominent troughs in the post-UV profiles in the *rnhA* and *rnhAB* mutants (Figs. 2B, 3C, and 4B)—clearly showing additional replication initiations all over the chromosome and inhibition of forks going toward the origin at the *rrn* positions. But it also highlighted an unexpected phenomenon—equal inhibition of the *oriK-3850*-initiated forks, which are co-directional to all these *rrn* operons. That is, UV-induced replication–transcription conflict inhibits forks equally well in both directions! For example, the most prominent replication profile discontinuity, which already appears at 60 min post-UV, is located between the UV-induced *oriK-3850* and *oriK-4300* and coincides with the *rrnCABE* cluster (Fig. 2B). The *rrnCABE* cluster appears almost impassable for post-UV replication in the *rnhAB* mutant, since the profile shape in this area does not change for up to 3 h (Fig. 3A)—however, indexation using the DNA accumulation data (Fig. 2C) reveals at least 6-fold more DNA there 180 min post-UV (Fig. 3C). This hard-to-replicate region between the two close dominant origins confirms that codirectional replication–transcription conflicts can be as severe as head-on conflicts. In fact, post-UV replication in the absence of both RNase H and RecBCD enzymes is essentially blocked at the (codirectional) *rrnG* and *rrnH* operons (Fig. 4B).

Therefore, we propose that, in the *rnhAB* mutants, codirectional replication–transcription conflicts at actively-transcribed genes, like *rrn* operons, involve formation of UV-induced RDH structures blocking replication through the region. Since at 60 min post-UV, the slopes of the profile in the origin macrodomain between the *rrnD* and the *rrnE* operons (covering ∼16% of the genome between coordinates 3426 and 4106 kb) are steep in all strains, including WT (Supplementary Fig. S2), while recovery of normal replication is slow and incomplete in the *rnhAB* mutant strains, we propose that after UV-exposure, the *rrn* operons, especially in the *rrnCABE* region, accumulate some RDH-containing structures, that stall both codirectional and head-on replication forks. Moreover, both the RNase H and RecBCD pathways are required to fully overcome these blocks. To address the critical role of *rrnCABE* region in this inhibition, we are planning to employ the *rnhAB* mutants with all *rrn* operons deleted from the chromosome.

**Figure 7.**
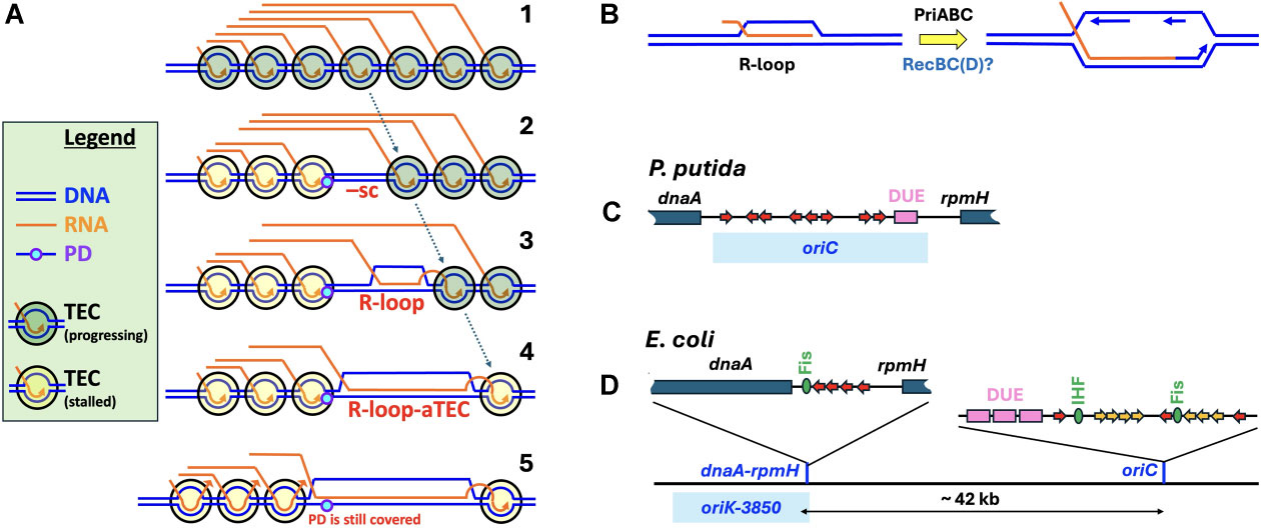
A model of UV-induced replication–transcription conflict and the nature of *oriK*s. (**A**) Our model of how an R-loop in the middle of a heavily transcribed region could make a PD irreparable and to stall the replication forks. The legend in the frame on the left explains the elements in the picture. TEC, transcription elongation complex; PD, pyrimidine dimer. (i) Normal transcription of an *rrn* operon; (ii) after UV, one of the TECs stalls on PD, while the growing naked DNA region between the stalled and the downstream-progressing TECs accumulates negative supercoiling (“–sc”); (iii) The accumulating –sc induces the formation of R-loop behind the last progressing TEC; (iv) the last moving TEC generates more –sc, enlarges the R-loop in R-loop-anchored TEC (R-loop-aTEC), and is stalled; (v) The stalled (by PD) TEC backtracking in an attempt to make PD accessible to NER is negated by R-loop spreading over the region. Note that return from “5” to “4” keeps the PD out of NER’s reach. (**B**) The assumed initiation at *oriK* zones from a transcription-generated R-loop. (**C**) A scheme of *Pseudomonas putida dnaA-oriC-rpmH* region, representing a typical bacterial replication origin (from [93]). Small red arrows indicate the orientation of DnaA-binding sites. DUE = DNA unwinding element. (**D**) A scheme of the *oriK-3850—oriC* chromosome region of *E. coli*, with the protein binding sites shown at the expanded *dnaA*-*rpmH* intergenic region and at the *oriC* proper. Small yellow arrows indicate weak DnaA-binding sites.

What could be the nature of UV-induced replication–transcription conflicts that (i) happen at heavily transcribed regions; (ii) block both head-on and codirectional forks; (iii) cannot be resolved in RNaseH deficient cells; (iv) are resolved without RNase H if TECs are destabilized due to the rpoB* defect? The following scenario in the continuously transcribed *rrn* operon envisions a TEC stalling in the middle, at a UV-lesion in the template DNA strand, while the preceding TEC eventually stalling downstream, due to the formation of a long R-loop-aTEC (Fig. 7A, steps 1→4). There is no known way for NER to get to the UV-lesion in this case, as it is either covered by the stalled RNAP (Fig. 7A, step 4) or, if RNAP backtracks, by the upstream spread of R-loop (Fig. 7A, step 5). The replication fork coming from the right to this region will be in the head-on orientation and therefore blocked by positive supercoiling [53], but why would a codirectional fork (coming from the left) be unable to displace the stalled TECs? The clue is provided by the absence of UV-induced replication–transcription conflict in the rpoB* mutants: prevention of the conflict by TEC instability means that replication forks in *E. coli* have no mechanisms of displacing co-directional TECs and must follow them through the whole gene until its end.

The evidence that *rrn* operons interfere with codirectional forks comes from ChIP-seq studies, reporting that *in vivo rrn* operons are associated with SMC condensin and with replicative helicase and fork restart proteins in *Bacillus subtilis* [44, 45], and with SeqA organizer of nascent DNA behind forks and with RecA recombinational repair enzyme in *E. coli* [46, 47]. However, these associations tend to be dismissed as artefacts, as no corresponding fork slowdown is obvious in (low-resolution) chromosomal profiles in any WT bacterium. Still, our high-resolution profiles of replicating WT cultures occasionally reveal a slower progress through the *rrnCABE* region (Supplementary Fig. S2, the log and 60 min profiles) or shallow troughs at the *rrn* operons (Supplementary Fig. S15B). To detect the slowing down of forks at the *rrn* locations, we looked for trendline discontinuities due to copy number downshifts (Supplementary Fig. S15A) in the WT profiles and indeed found at least four such downshifts, indicative of fork stalling (Supplementary Fig. S15B). One discontinuity—at *terA*—was expected and served as a positive control for the procedure. The other three were either at *rrnG* or *rrnD* directly, or downstream of the *rrnCABE* cluster; in fact, only *rrnH* caused no apparent downshift in the WT *E. coli* replication patterns (Supplementary Fig. S15B). On the basis of this preliminary analysis, we suggest that ribosomal operons in *E. coli* do slow down co-directional replication forks—meaning that forks have no mechanism of displacing co-directional TECs and simply follow them at a 10-times slower rate through heavily transcribed genes. And in the case of R-loop-aTEC formation around PD in the RNase H-deficient cells, the resulting RNAP cavalcade may be indeed stuck with no possibility of formal resolution (Fig. 7A), but at the same time blocking replication in both directions.

### The nature of random initiation in the absence of RNase HI

Why would *rnhA* mutants initiate all over the chromosome? This is due to a combination of the presumed accumulation of R-loops (detected as RDH signal) in the chromosomal DNA in the absence of RNase HI enzyme [17, 24] and a close resemblance between R-loops and the recombinational repair intermediates in the pathway to restore disintegrated replication forks [54]. When a replication fork disintegrates (as a result of various scenarios, like collapse, regress-split, rear-ending or a DSB behind [62, 76, 83]), the replication fork framework is reassembled using recombinational repair [8]. In bacteria, the R-loop-like product of this reassembly, the strand-invasion intermediate, is recognized by PriABC enzymes that load the replisome back onto the fork framework to restart the fork [84]. In a similar scenario, any persistent R-loop could start a replication bubble, via PriABC-dependent loading of the replisome (Fig. 7B) [54].

These events should be generally rare, but since the number of R-loops in the *rnhA* mutant is several times higher than in WT, the overall chromosome-wide rate of initiation from them may be enough to suppress scheduled initiations from *oriC*. Interestingly, if initiations from R-loops are random, while *oriC* firing is suppressed, then the resulting overall profile is expected to be flat. Indeed, the *rnhA* mutant profile *is* flatter than the WT one (Fig. 1), but it is still higher around the origin and lower around the terminus. The failure to flatten the profile completely could reflect the existence of several transcription-related gradients along the replichores in the *E. coli* chromosome, with the highest point in the origin macrodomain and the lowest point around the terminus: for rRNA operons and highly-expressed proteins, for sigma-70-regulated promoters (= growth genes where R-loops may form), for the gyrase binding sites and therefore the inferred negative supercoiling (which stabilizes R-loops), for the local transcription propensity [85–87]. These gradients should ensure that random initiations from R-loops would preserve the same high-in-the-origin, low-in-the-terminus profile.

Relative to the *rnhA* single mutants, the more severe growth and replication defect of the *rnhAB* double mutants is reflected in their higher SOS-induction [18], so some local *oriK*s could be potentially induced by nearby SOS genes. Decreasing the span parameter for LOESS averaging in our high-resolution profiles (to show more details), we related the position of all known SOS-induced promoters with the local peaks and troughs of the LOESS curves of the 60 min post-UV profile and the logarithmic profile (Supplementary Fig. S16, only the post-UV profiles are shown). Interestingly, the only UV-induced peak in this strain was *oriK-3850* (Fig. 3B and Supplementary Fig. S16) (also, see below); the other peaks/bumps and troughs were all present before UV. We found that, out of 26 LexA-controlled promoters (regulating some 30 SOS genes), 17–18 could be (subjectively) associated with local profile peaks, while only 2–6 are associated with local profile troughs (Supplementary Fig. S16), consistent with the idea that overexpression of genes of the SOS regulon is behind some local *oriK*s. We also noticed that the vegetative *oriK-4300* of the *rnhA* mutants could be itself driven by bona fide SOS genes nearby, *lexA* (4257 kb) and *uvrA* (4274 kb) (Fig. 3B and Supplementary Fig. S16). However, testing whether deletion of *lexA* together with its promoter affects the strength of *oriK-4300* produced no strong or consistent effects (Supplementary Fig. S17).

### The nature of *oriK* zones and the variable strength of the *ter* sites

The discussion above implies no special initiation sites in the *rnhA* mutant cells, but at the same time we provide chromosome coordinates of peak initiations from *oriK* zones, which correlate well with *oriK* coordinates from other studies (Supplementary Table S2). In fact, researchers of the *rnhA* mutants would always speak about specific *oriK*s [24, 26, 27, 55]. At the same time, *oriK*s were never mapped precisely, while attempts at their cloning by asking them to drive plasmid replication also failed [56, 57], for no apparent reason.

Our high-resolution replication profiles provide a possible answer: there may be no specific initiation sites in any given *oriK* zone—just a uniformly-low initiation potential throughout the zone. Instead of specific initiation peaks, there are specific chromosomal sites that *inhibit* replication—and these serve as separators that define the limits of *oriK* zones (Fig. 1). In WT cells, replication fork inhibition is the function of the two unidirectional termination sites, *terA* and *terC*, that trap replication forks within the termination zone, which these two sites bracket (Fig. 1A). But even these two *ter*-sites do not reveal themselves during unchallenged growth of WT cells, generating an artificial “origin in the terminus” only in cells recovering from DNA damage [70] (Supplementary Fig. S2).

The other eight consensus *ter* sites [88, 89] positioned various distances from the termination zone—four in the right replichore blocking CCW forks, while another four in the left replichore blocking CW forks (Fig. 1A and Supplementary Table S1)—never reveal themselves in WT cells. In contrast, even during unchallenged growth in the *rnhA* mutants, random initiations reveal some of these *ter* sites (Fig. 1E), that start serving as additional “delineators” throughout the terminus-half of the chromosome. Interestingly, this provides an *in vivo* test for the functionality of individual termination sites in the *E. coli* chromosome. Before, the strength of these sites had to be compared either in plasmids [88] or *in vitro* [89], both approaches showing *terF* as the weakest site. Our profiles (e.g. Supplementary Fig. S16) show no replication-inhibition activity at *terH* (599), *terI* (625), *terE* (1081), *terF* (2316 kb) and *terJ* (2574 kb); with the surprising exception of *terE*, our results (summarized in Supplementary Table S1) are consistent with the recent re-classification of these as pseudo-*ter* sites, as showing no Tus protein binding *in vivo* [90].

In addition to *ter* sites in the *rnhA* mutants, the *rrn* operons are hard-to-replicate points, becoming natural delineators in the origin-half of the chromosome. Even the *oriC* location seems hard-to-replicate in the absence of RNase HI (Fig. 1D) (unless it is an artefact of proximity to the *rrnC* operon). The origin-proximal *rrn* operons are the reason that there are three *oriK* zones in the *oriC* macrodomain instead of one. The high-resolution profiling suggests that it could have been a single initiation zone—yet it is cut into three “peaks” by the *oriC-rrnC* trough on the left and by the *rrnBE* trough on the right (Fig. 1D). In the bacterial chromosomes, the *rrn* operons are always oriented away from *oriC* [91], as first noticed in *E. coli* (Fig. 1A) and explained in terms of the avoidance of head-on replication–transcription conflict [40]. Remarkably, migrating the main initiation point to *oriK-4300* in the *rnhA* mutants makes its leftward forks meat the *rrnCABE* cluster head-on (Fig. 1D), which should inhibit them. This is probably why in some runs in various *rnhA* mutants, the *oriK-3850* and *oriK-4100* help sustain replication in this region and in the left replichore in general (Fig. 1C and Supplementary Fig. S1B).

### The inducibility of *oriK-3850*

While most *oriK* zones in the *rnhA* mutants appeared insensitive to change in conditions, two conditional *oriK* phenomena were revealed by UV-irradiation. First, *oriK-3850* is UV-induced in all our *rnhA* mutants except when they also have the *dnaA* defect. Initially, *oriK-3850* attracted our attention because of its proximity to *oriC*; in fact, when we started with low-resolution LOESS, we thought this *oriK* initiation zone *was* at *oriC*, while the leftward shift was an artifact of averaging. The unique *oriC* in bacterial chromosomes comprises several binding sites of the DnaA initiator protein, typically in clusters around the *dnaA* gene itself, which is thus self-regulated via its promoter-associated cluster [92, 93] (Fig. 7C). The *oriC* of *E. coli* is atypical in that it is found ∼42 kb away from the *dnaA* gene, which means that there is a separate cluster of DnaA-boxes in the *dnaA* promoter for self-regulation [94, 95] (Fig. 7D). Remarkably, our high-resolution profiling in the *rnhAB* mutants revealed inactivity of *oriC* proper, compensated by the initiation activity of the nearby *oriK-3850* (zone 5 in Fig. 1C) which borders *dnaA*.

Interestingly, initiation from *oriK-3850* instead of *oriC* makes this new chromosomal origin symmetrical about the major ribosomal operons, *rrnG* and *rrnD* to the left versus *rrnE* and *rrnH* to the right (Fig. 1A and C). Could the activity of *oriK-3850* downstream of the *dnaA-dnaN-recF-gyrB* operon and especially its robust UV-induction be linked to SOS (see above) or perhaps to an even broader transcriptional response to DNA damage? The *dnaA* promoter has no LexA binding site, and although mildly induced after UV, this induction is still observed in the *lexA1* (IND) mutant [16]. Therefore, it is formally possible that the SOS-independent increase in transcription of the *dnaA* operon drives *oriK-3850* initiations after DNA damage; this idea could be tested by moving the *dnaA* gene to a different chromosomal location or to a plasmid.

Assuming that the DnaA boxes at the *dnaA* promoter were important for initiation from *oriK-3850*, the lack of UV-induction of *oriK-3850* in the *rnhAB dnaA* mutant was almost expected, while the surprise came as the importance of the *oriC* site for the full *oriK-3850* induction. If DnaA polymerizes to form a multi-subunit platform on the nucleoid surface, as suggested for other proteins that polymerize on DNA [96], it is possible that both DNA regions, *oriC* and *oriK-3850*, simultaneously interact with this DnaA platform, and *oriC*, instead of initiating itself, somehow helps to initiate at *oriK-3850*. We were lucky that the *rnhAB* mutants switch initiations from the *oriC*-distal location (*oriK-4300*) to *oriC*-proximal *oriK-3850* during post-UV recovery—thus providing a clear evidence for the DnaA-dependent “UV-induced *oriC* firing”—contrasting with the reported lack of UV-induced *oriC* firing in WT cells [97]. When post-UV replication recovers (in the *rnhA* and rnhAB rpoB* mutant), initiation switches back from *oriK-3850* to *oriK-4300*. In the light of our negative Δ*lexA* result (Supplementary Fig. S17), the nature of initiations at *oriK-4300* remains a mystery.

The second UV-sensitive initiation phenomenon was observed in the *rnhAB recBC*(Ts) mutant. We expected replication activity to subside throughout the chromosome in the *rnhAB recBC*(Ts) mutant at the non-permissive temperature, but instead observed that only the origin-half of the chromosome was subsiding (slowly), while the terminus-half kept being active and even appeared initiating more (Fig. 4A), as if some types of cSDR initiations are RecBCD-independent. We did not follow this further. The real surprise was that, after UV irradiation, the initiation pattern of the whole *rnhAB recBC* mutant chromosome flipped: now the initiations were essentially restricted to the origin-half of the chromosome, with the major initiations happening at *oriK-3850* (Fig. 4B). Thus, paradoxically, initiations in the terminus-half of the chromosome are RecBCD-independent without UV, but they become RecBCD-dependent after UV! Perhaps, this is the strongest and the most puzzling effect of UV on chromosome replication patterns that we have ever observed.

## Supplementary Material

gkaf282_Supplemental_Files

## Data Availability

The data underlying this article are available in NCBI’s Sequence Read Archive (at https://www.ncbi.nlm.nih.gov/sra) and can be accessed as BioProject PRJNA1220109.
