# Supplementary material for: UV induces codirectional replication–transcription conflicts and an alternative DnaA-dependent replication origin in the rnhAB mutants of Escherichiacoli
Source: Nucleic Acids Res. 2025 Apr 16;53(7):gkaf282. doi: 10.1093/nar/gkaf282 (PMC12000880; doi:10.1093/nar/gkaf282)
Supplement: gkaf282_Supplemental_Files [file gkaf282_supplemental_files.zip › Kouzminova — Supplement.pdf]

Supplement for the paper

UV induces codirectional replication-transcription conflicts and an alternative DnaA-dependent replication origin in the *rnhAB* mutants of *E. coli*

by Elena A. Kouzminova, Glen E. Cronan and Andrei Kuzminov

Supplemental figures

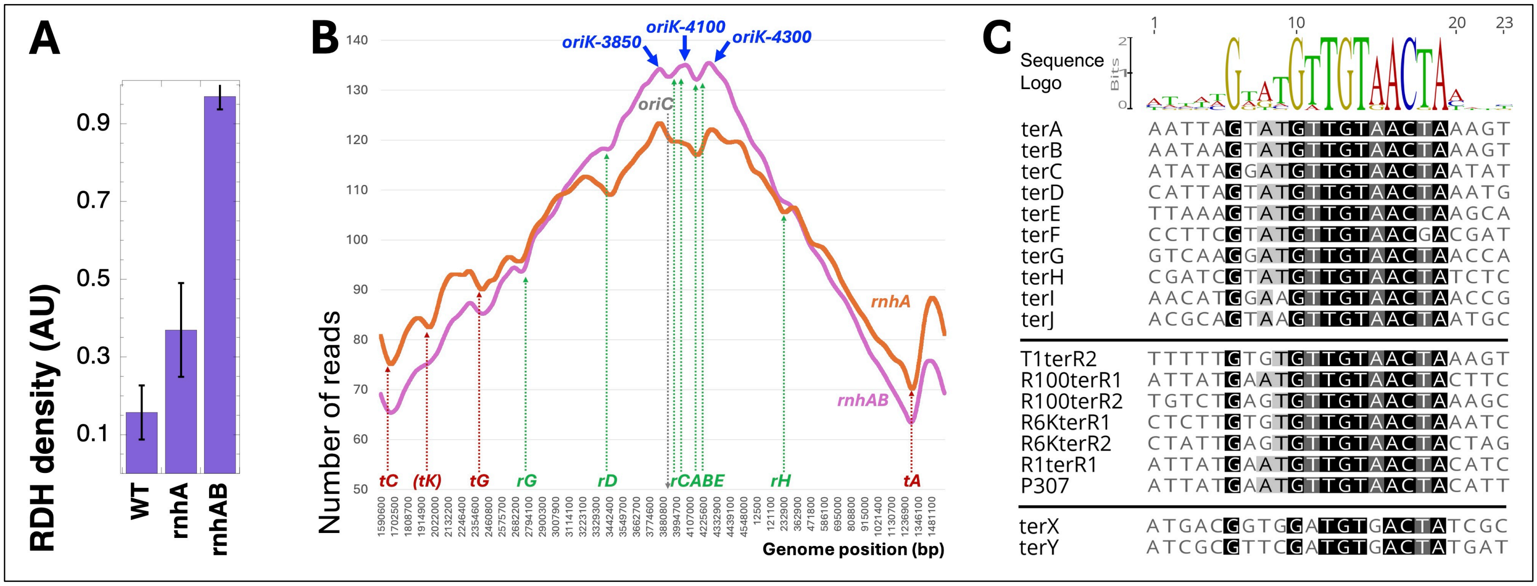

Fig. S1. Explanation of some unexpected features of the *rnhA* mutant profiles.

A. RDH signal density (in arbitrary units) in the genomic DNA of rapidly growing WT (AB1157), *rnhA* (L-413) and *rnhAB* (L-416) strains. The values are modified from (Kouzminova and Kuzminov 2021).

B. Comparison of the *rnhA* profile with the averaged *rhnAB* profile. Strains: L-413 and L-416. The chromosome features causing troughs are listed at the bottom (including the proposed new *ter* site — *tK*). The three *oriK* zones in the origin macrodomain are marked on top. The averaged *rnhAB* replication profile is derived from five replication runs (see Methods) and is the top profile in Fig. S5.

C. The sequence logo of the replication termination site in *E. coli*, with the blocking direction from left to right. The 10 chromosomal sites are shown at the top, the 7 plasmid sites are in the middle. At the bottom are the two new proposed sites, *terX* (1,987,836 -> 1,987,813) and *terY* (2,052,133 -> 2,052,156), the two close sites that make up "*terK*". Located appropriately to explain the observed replication inhibition, the two sites are oriented in the opposite way on the chromosome, so the pair should be able to inhibit forks coming either way.

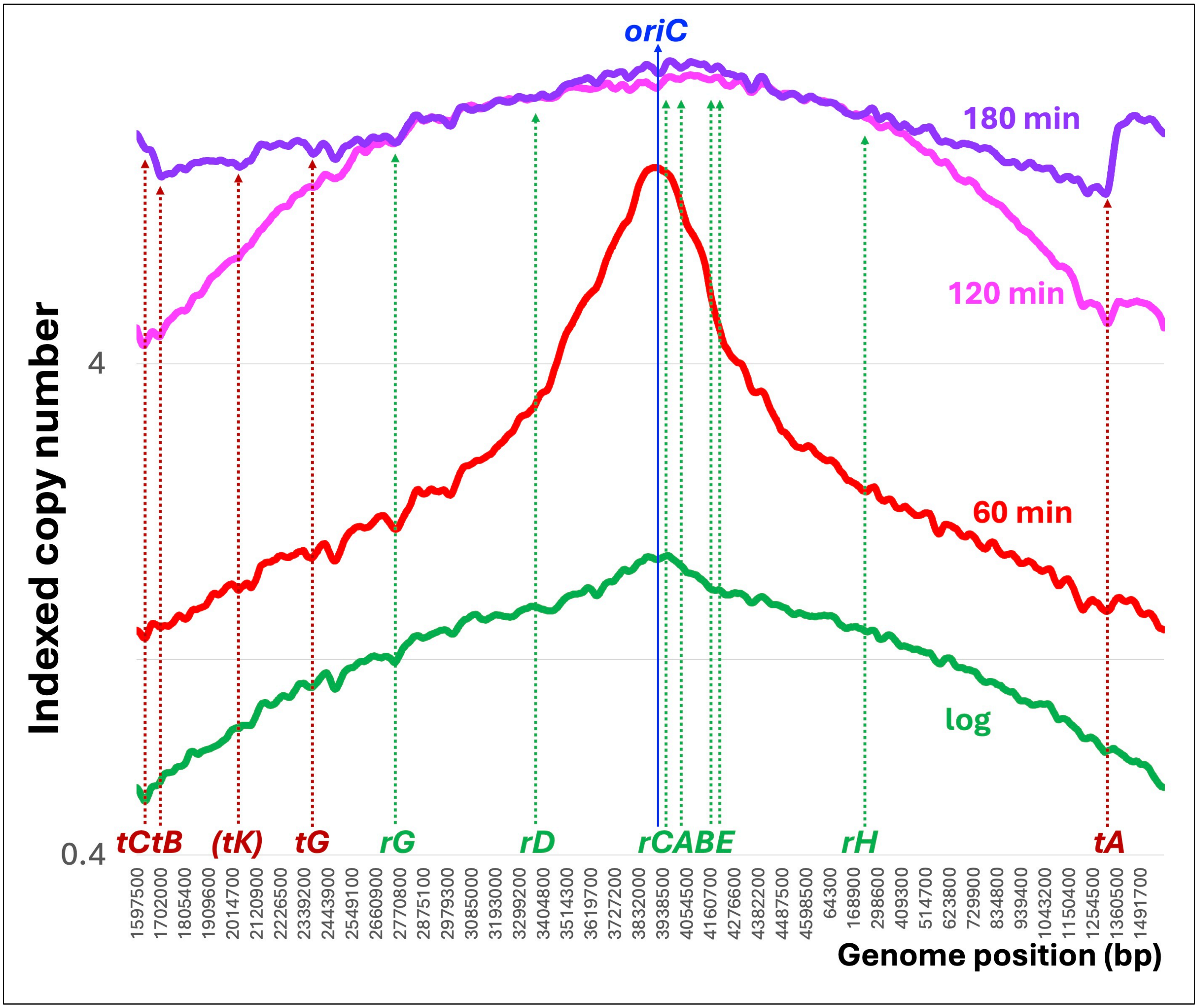

Fig. S2. Recovery of the post-UV replication in WT cells (this nested set will be fully described elsewhere). The Y-axis is in the log scale. The strain is AB1157.

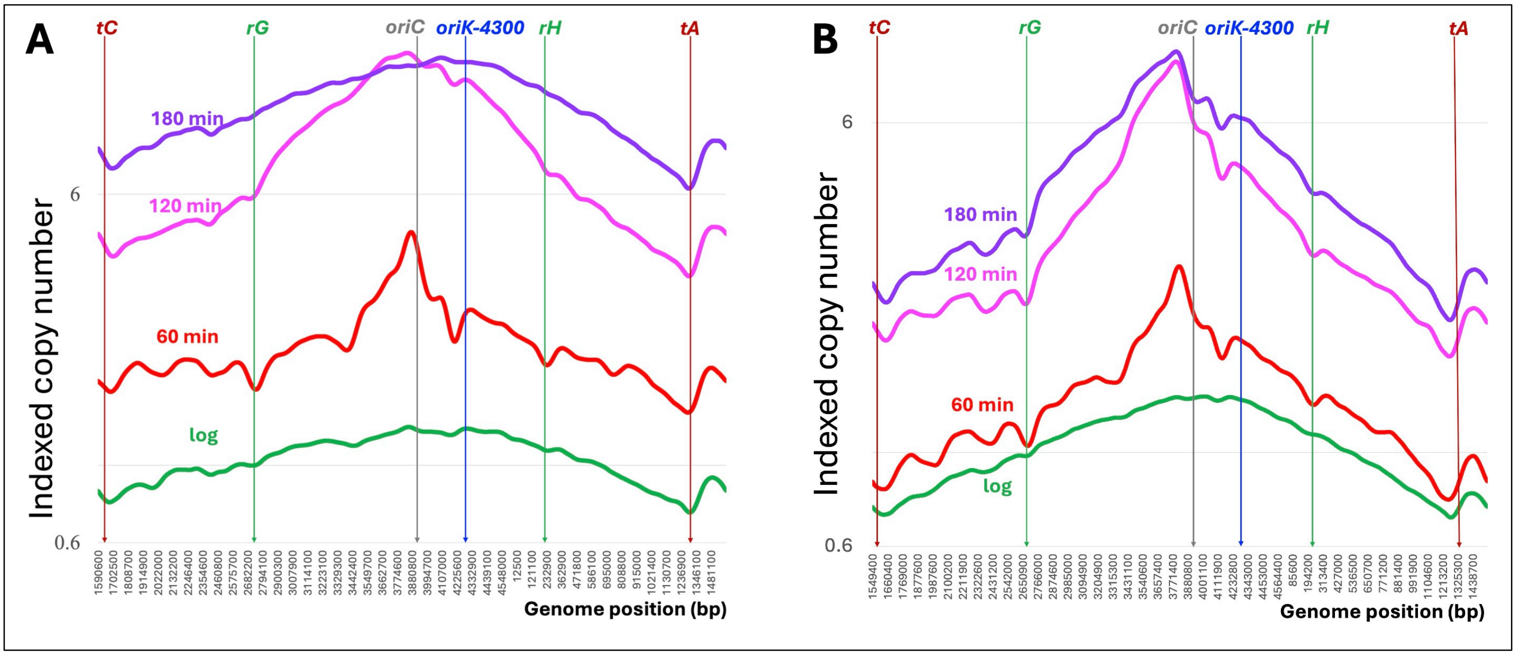

Fig. S3. Comparison of the nested profile sets for the *rnhA* single vs *rnhAB* double mutants.
The Y-axis is in the log scale.
A. The *rnhA* single mutant (the strain is L-413).
B. The *rnhAB* double mutant (the strain is L-416).

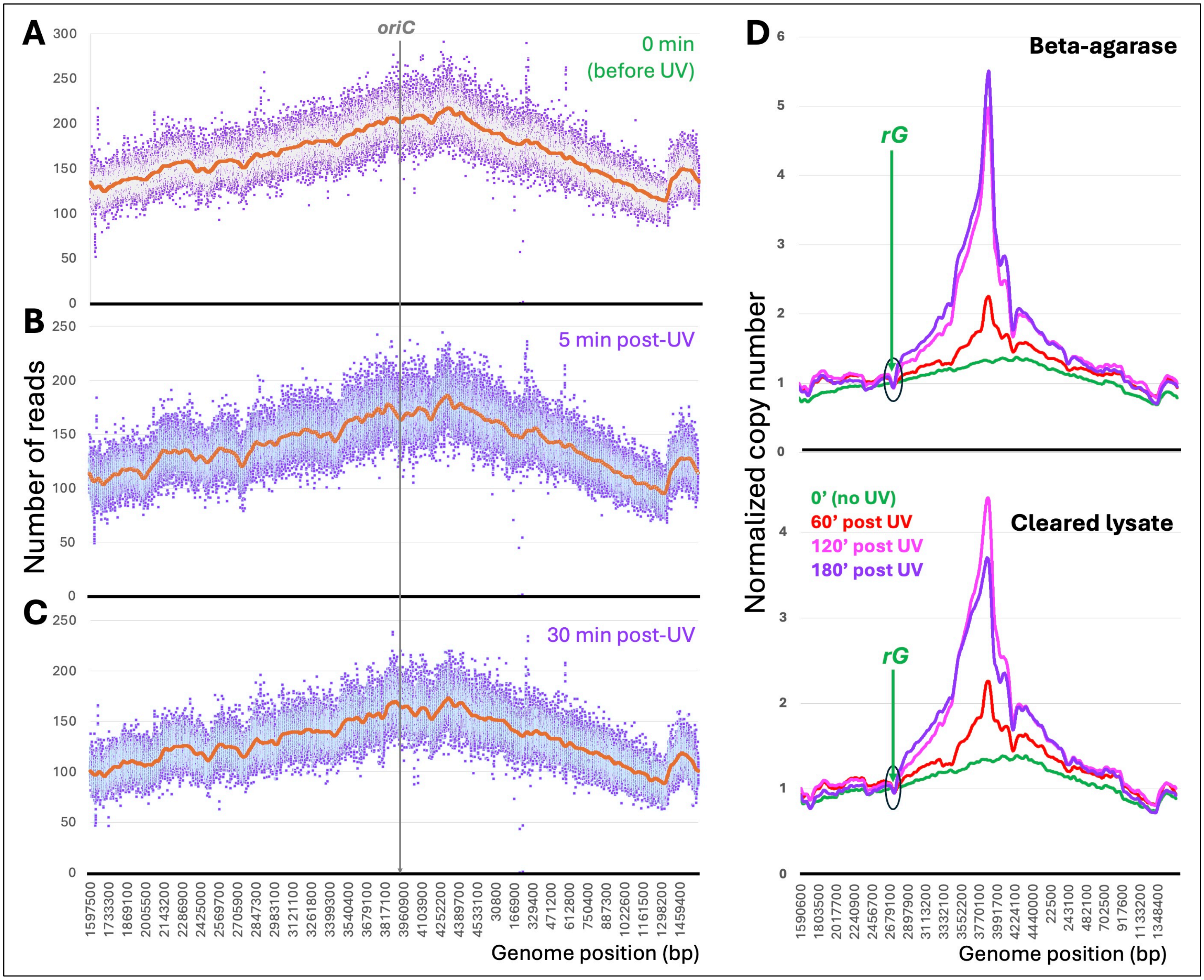

Fig. S4. The complete chromosomal profiles of the *rnhAB* mutant before UV (t=0) versus 5- and 30 min post-UV, as well as comparison of replication profiles after two DNA isolation protocols. In panels A, B and C, the *oriC* position is marked to reveal the UV-induced initiation activity there. The strain is L-416.

A. The logarithmic growth profile (from Fig. 3A, top).
B. The 5 min post-UV profile.
C. The 30 min post-UV profile.

D. Comparison of the two methods of total DNA isolation: isolation from agarose plugs by hydrolysis with beta-agarase versus the standard cleared lysate / phenol extraction procedure. LOESS profiles are normalized to the *rrnG* region (circled), like in Fig. 2B.

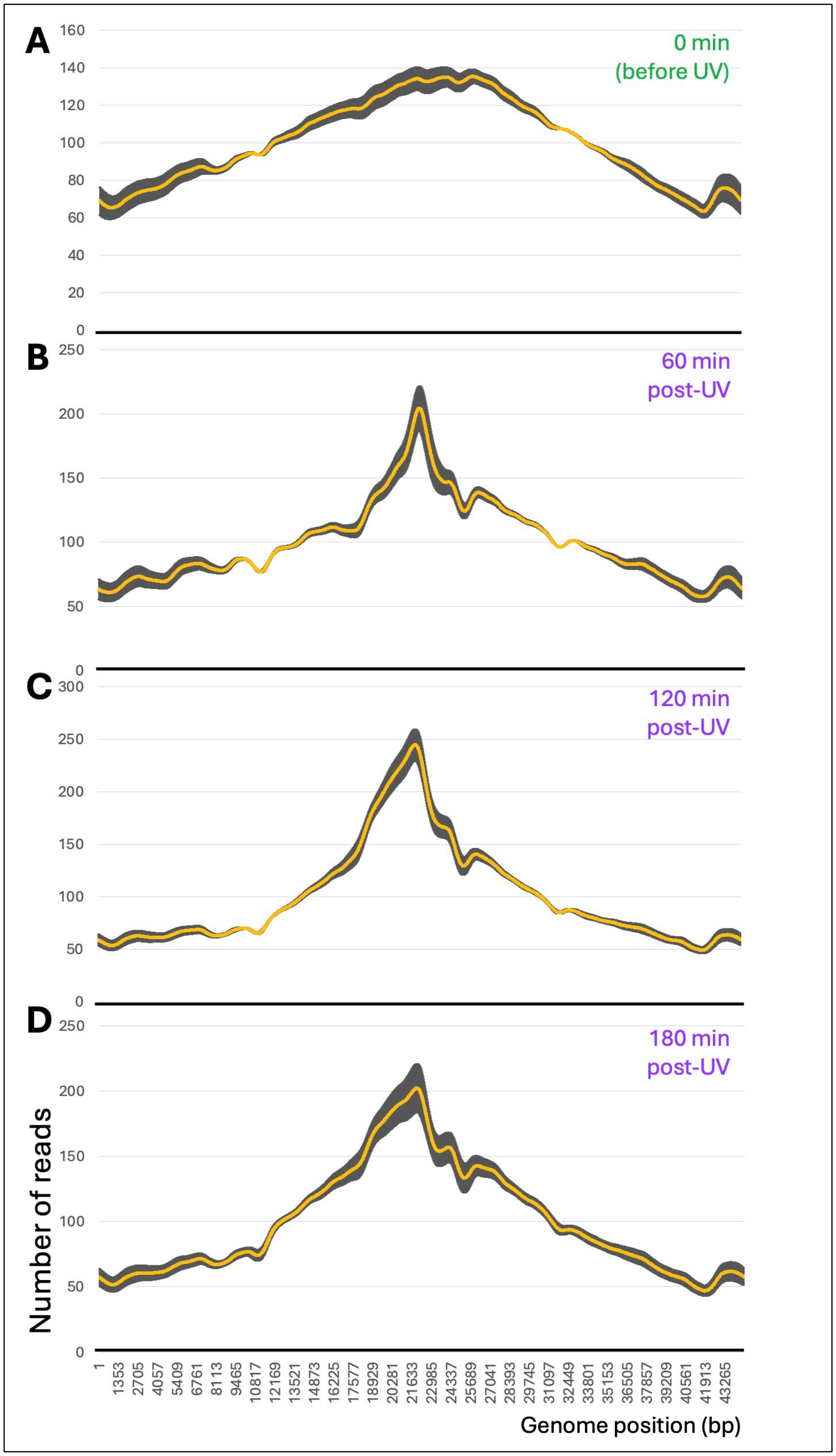

Fig. S5. Reproducibility of the *rnhAB* profiles of Fig. 3A. The means of LOESS values from five independent repetitions are shown, normalized to the average counts of their runs ± SEM (dark shadows). To generate these plots, first, binned per-base read depths were expressed as a percentage of the average bin read-depth within individual runs. LOESS trendlines (Materials and Methods) were then generated on this read-depth normalized data, and the LOESS values derived from individual biological replicates were used to calculate the mean and SEM error bands.

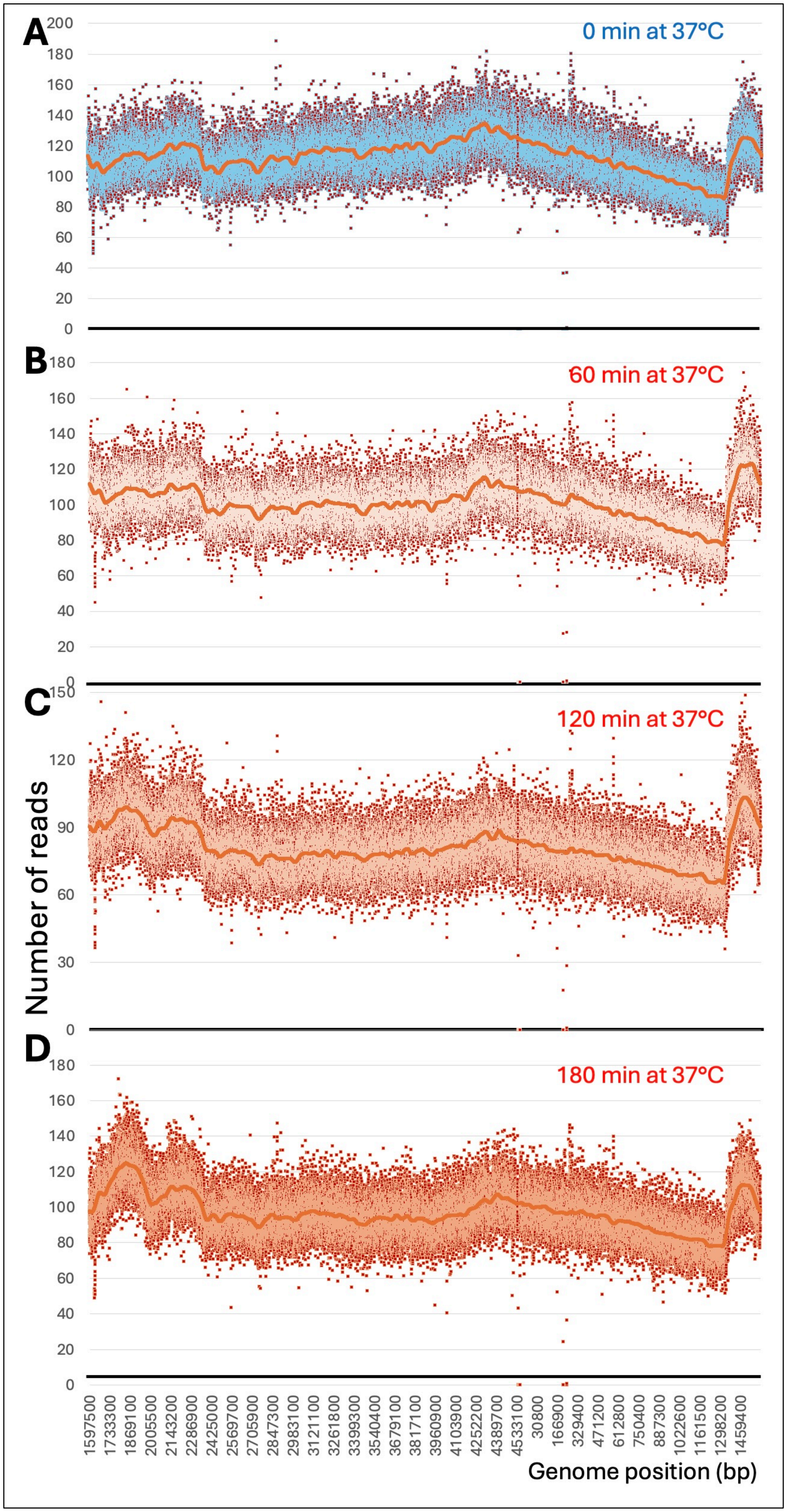

Fig. S6. Complete replication profiles of the *rnhAB recBC* mutant, switched from 28°C to 37° at time=0. The strain is L-476.

A. Just before the temperature switch (still at 28°C).

B. 60 min after the switch.

C. 120 min after the switch.

D. 180 min after the switch.

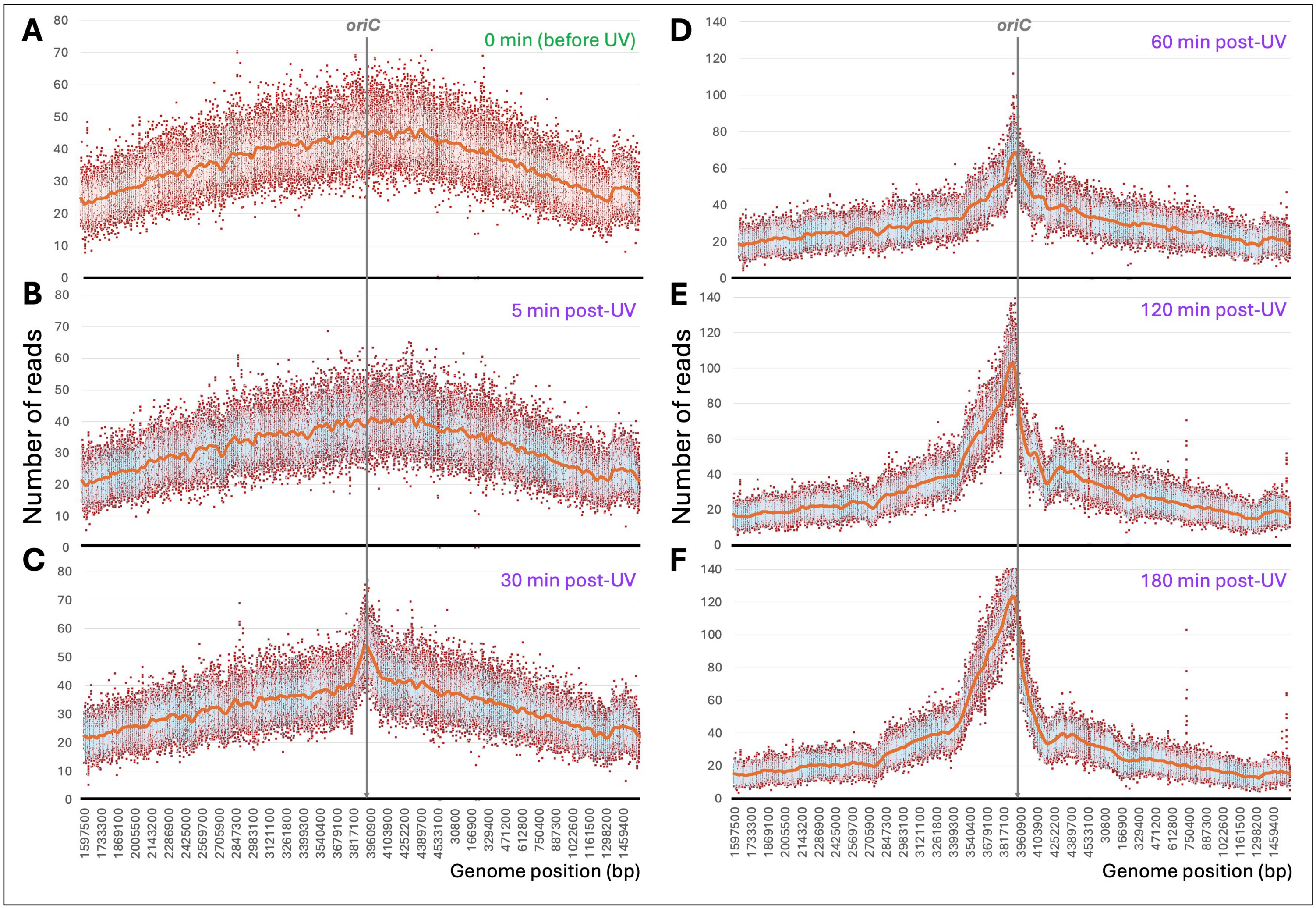

Fig. S7. Post-UV replication profiles of the *rnhAB recBC* mutant. The strain is L-476. After UV at time=0, the culture was shaken at 37° C for the indicated amount of time.

A. Before UV.
B. 5 min after UV.
C. 30 min after UV.
D. 60 min after UV.
E. 120 min after UV.
F. 180 min after UV.

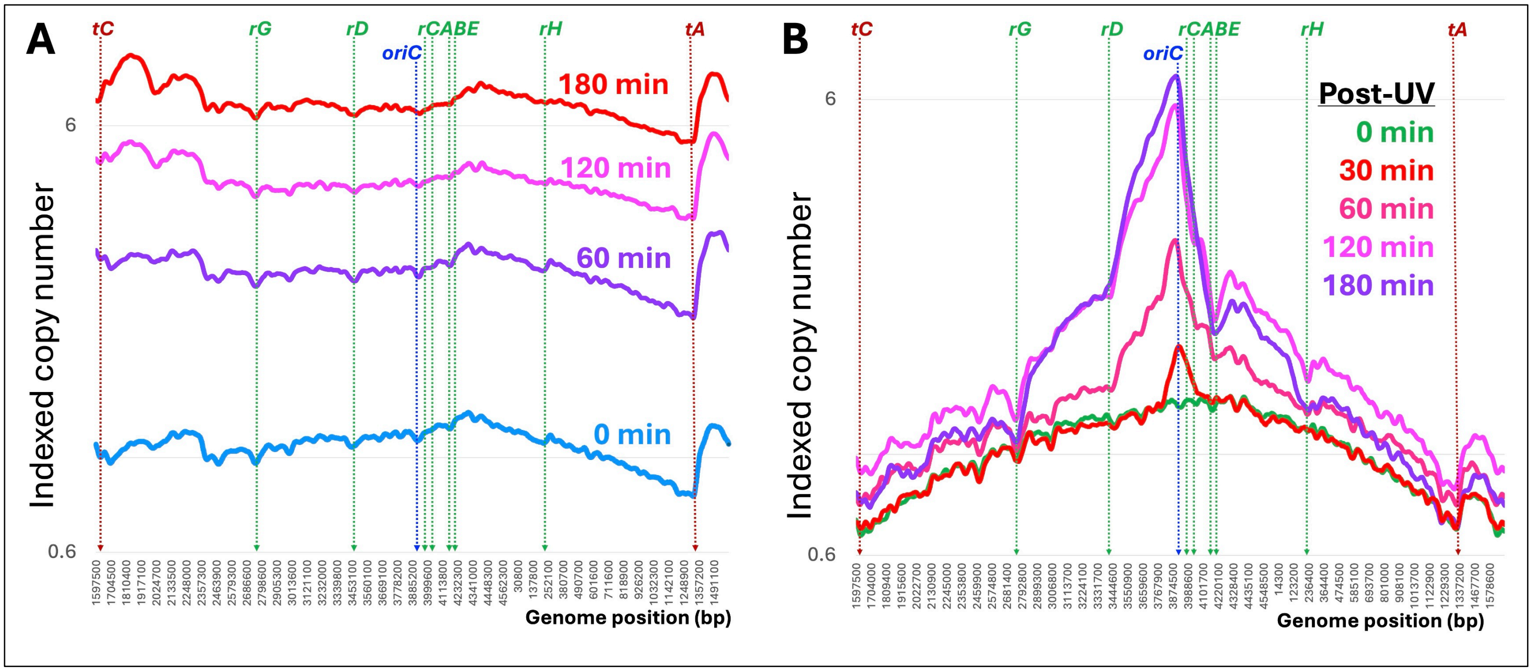

Fig. S8. The Y-Log versions of the nested sets of the *rnhAB recBC* mutant. The strain is L-476.
A. The unchallenged replication at 37°C.
B. Replication at 37°C after UV. For additional details see the legend to Fig. 4.

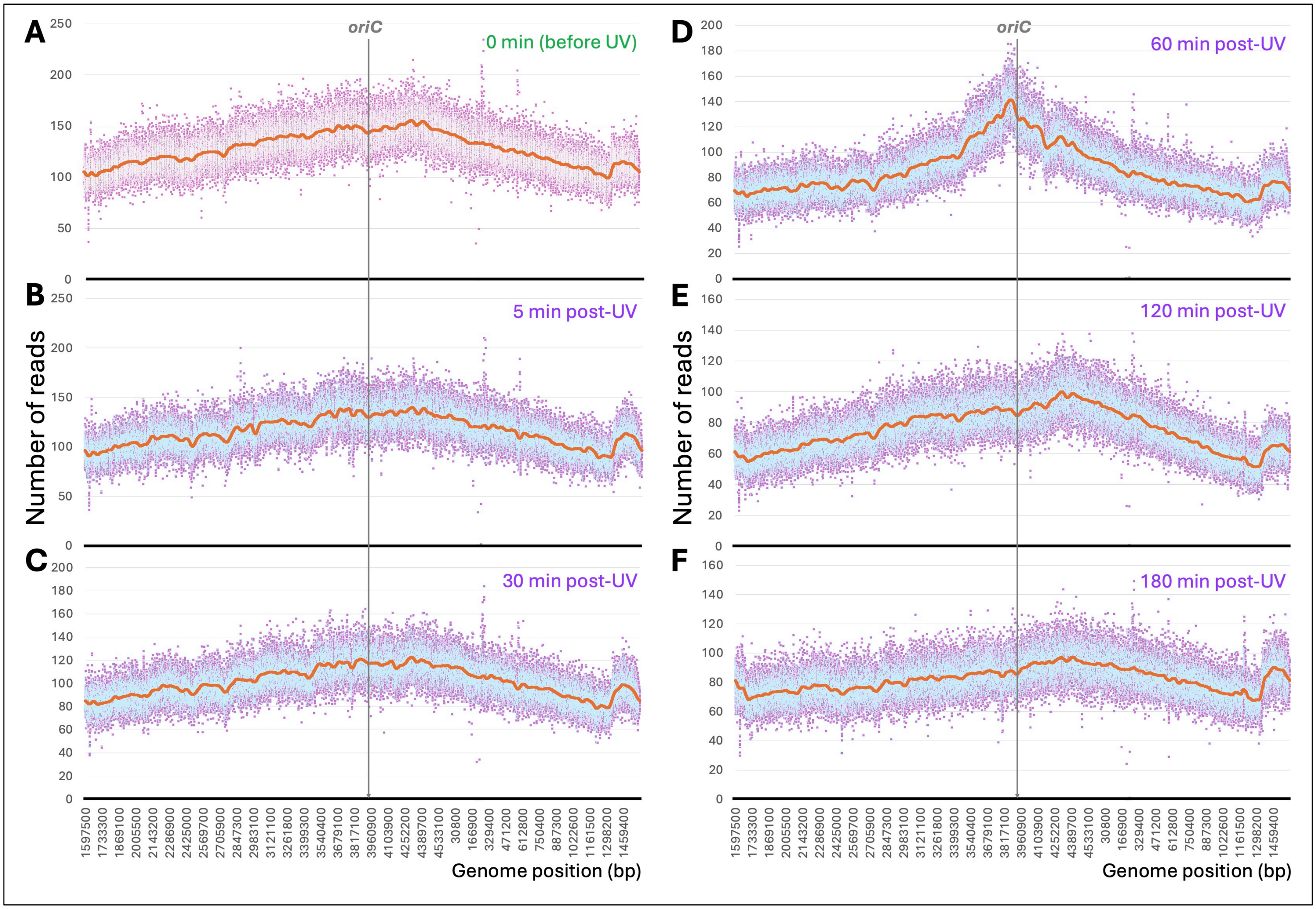

Fig. S9. The post-UV replication profiles of the *rnhAB rpoB mutant.** After UV at time=0, the culture was shaken at 37° C for the indicated amount of time. The strain is L-416-33.

A. Before UV.
B. 5 min after UV.
C. 30 min after UV.
D. 60 min after UV.
E. 120 min after UV.
F. 180 min after UV.

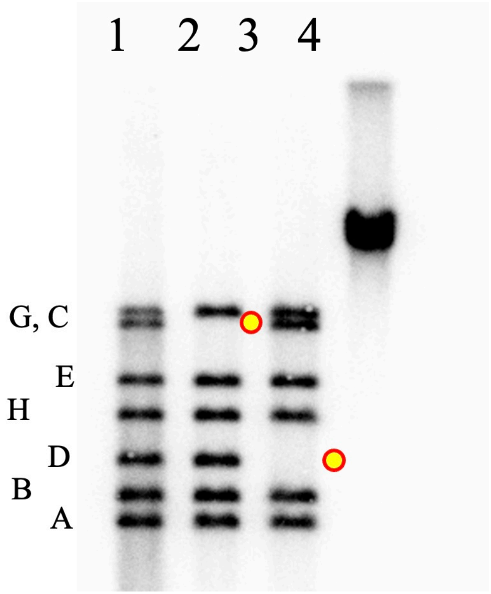

rrn operon	Fragment sizes (Bam H, PstI), bp
rrnC	16908
rrnG	15451
rrnE	11152
rrnH	9598
rrnD	8082
rrnB	7200
rrnA	6134

Fig. S10. Confirmation of specific *rrn* deletions. This analysis is based on (CONDON *et al.* 1993). Southern blot analysis of the BamHI-PstI digest of the chromosomal DNA from the Δ*rrnG* (L-527) and Δ*rrnD* (L-529) mutants. The ^{32}P-labelled probe comprised two PCR fragments*: rrsA (*0.9 kb) and *rrlA (*1.5 kb). Sizes of the expected *rrn* operon fragments are presented in the table; the missing bands due to specific deletions are indicated by red circles. Loading: 1, AB1157; 2, L-527; 3, L-529; 4, undigested chromosomal DNA of L-527. Specific *rrn*-operon bands are indicated by letters.

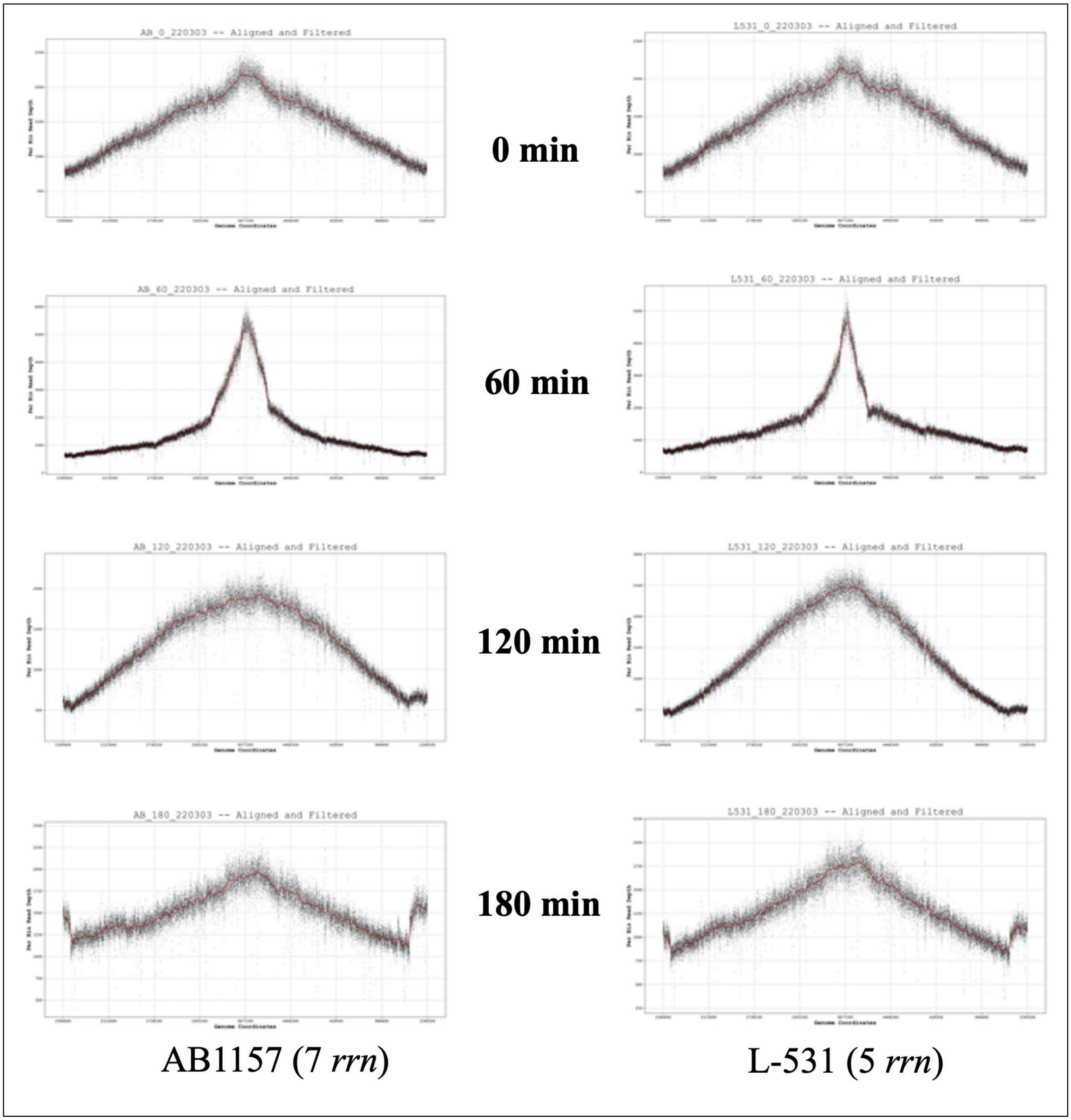

Fig. S11. Post-UV replication profiles of the WT strain with 7 *rrn* operons (AB1157)) and its derivative with 5 *rrn* operons (L-531-2). UV dose was 40 J/m^2. Absolute read counts (of 100 bp bins, dots) and LOESS values (brown curves) are plotted against genomic coordinates.

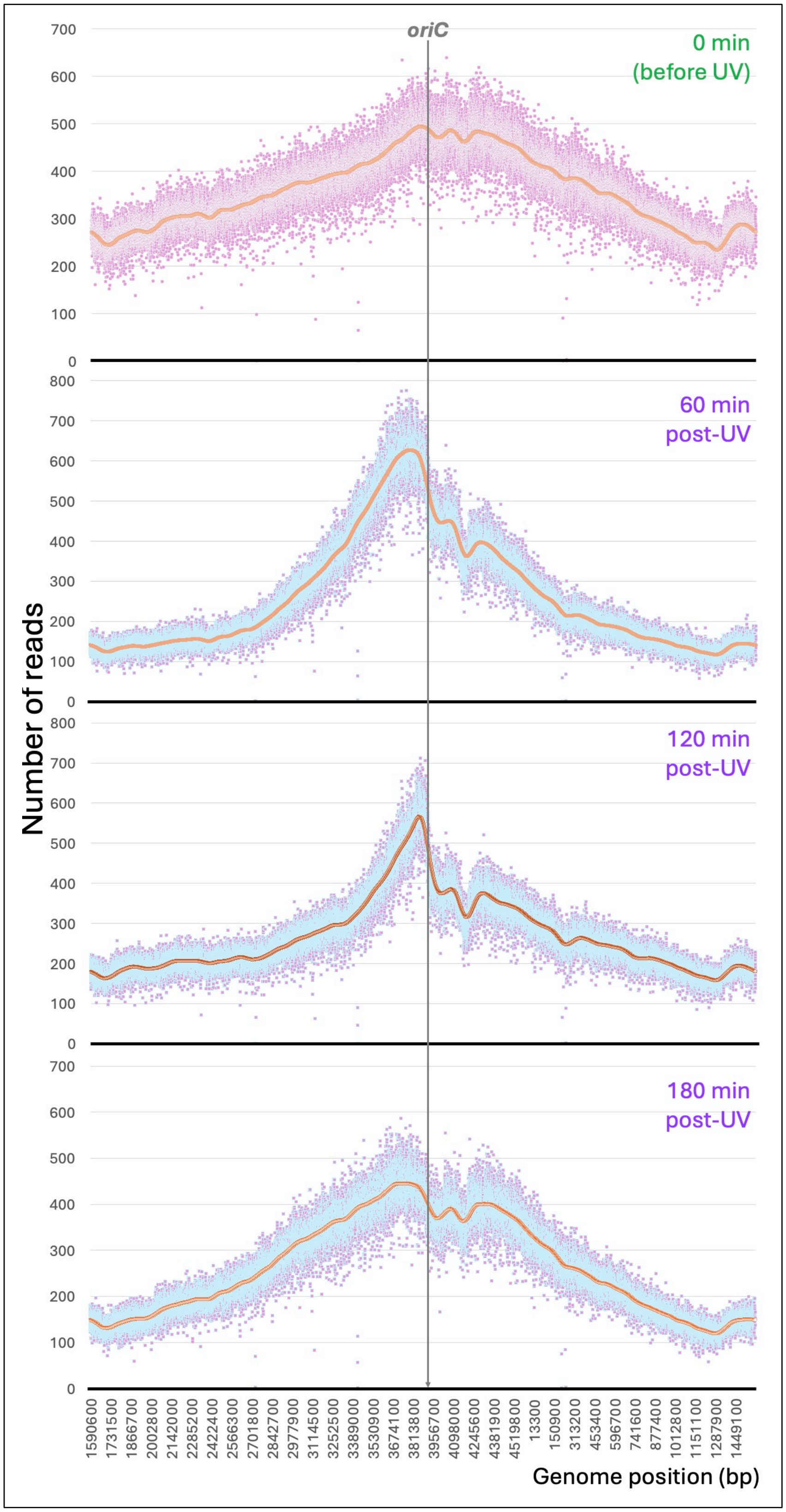

Fig. S12. Post-UV replication profiles of the *rnhAB* 5rrn mutant. The strain is L-534-2. After UV at time=0, the culture was shaken at 37° C for the indicated amount of time.

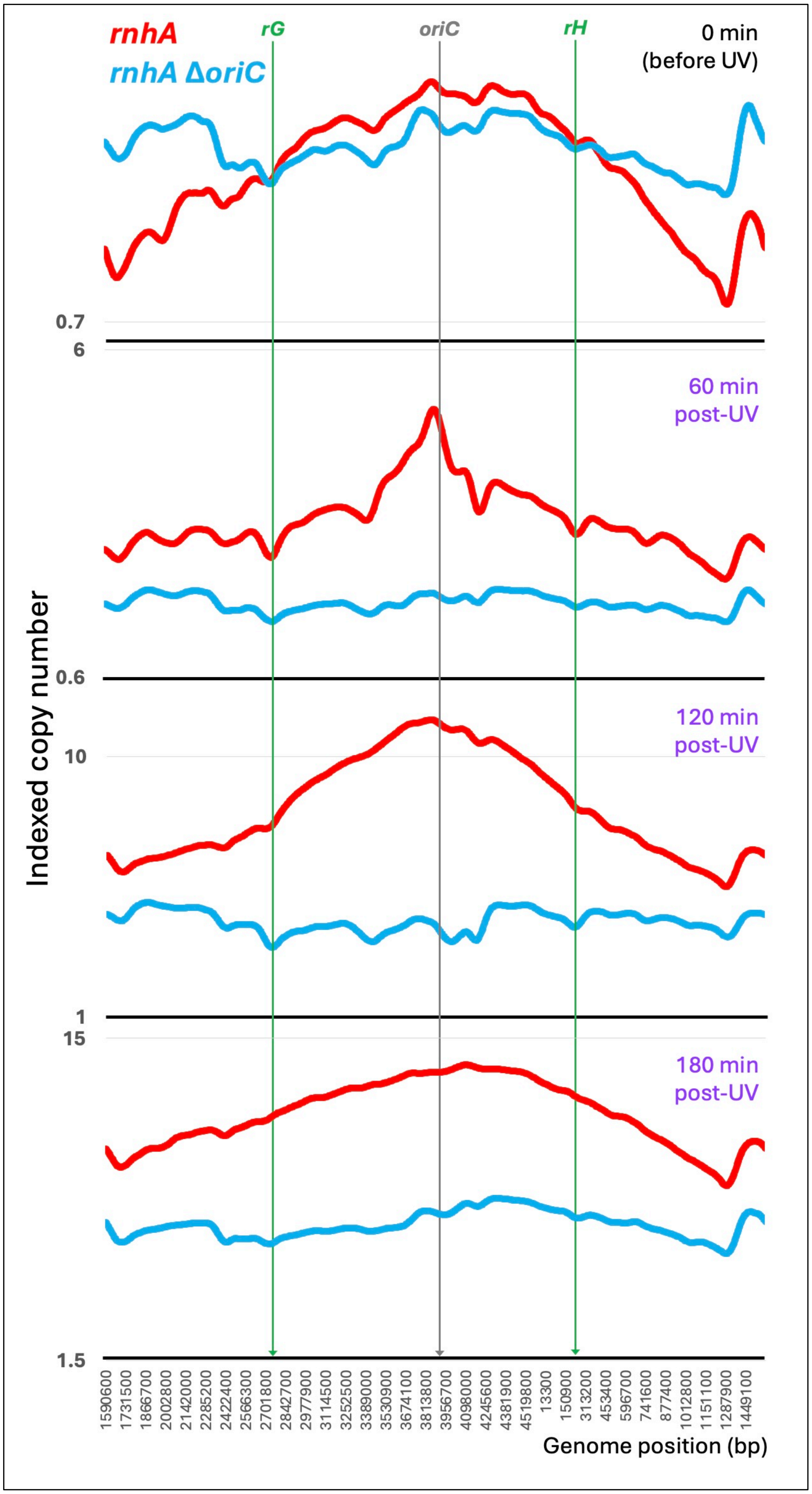

Fig. S13. Comparison of post-UV chromosomal replication of the *rnhA* single mutant vs *rnhA* Δ*oriC* mutant by time points using the indexed copy number profiles (Fig. 6C vs 2D). Y-axes are logarithmic, to show the details of the Δ*oriC* profiles. The strains are L-413 and L-555.

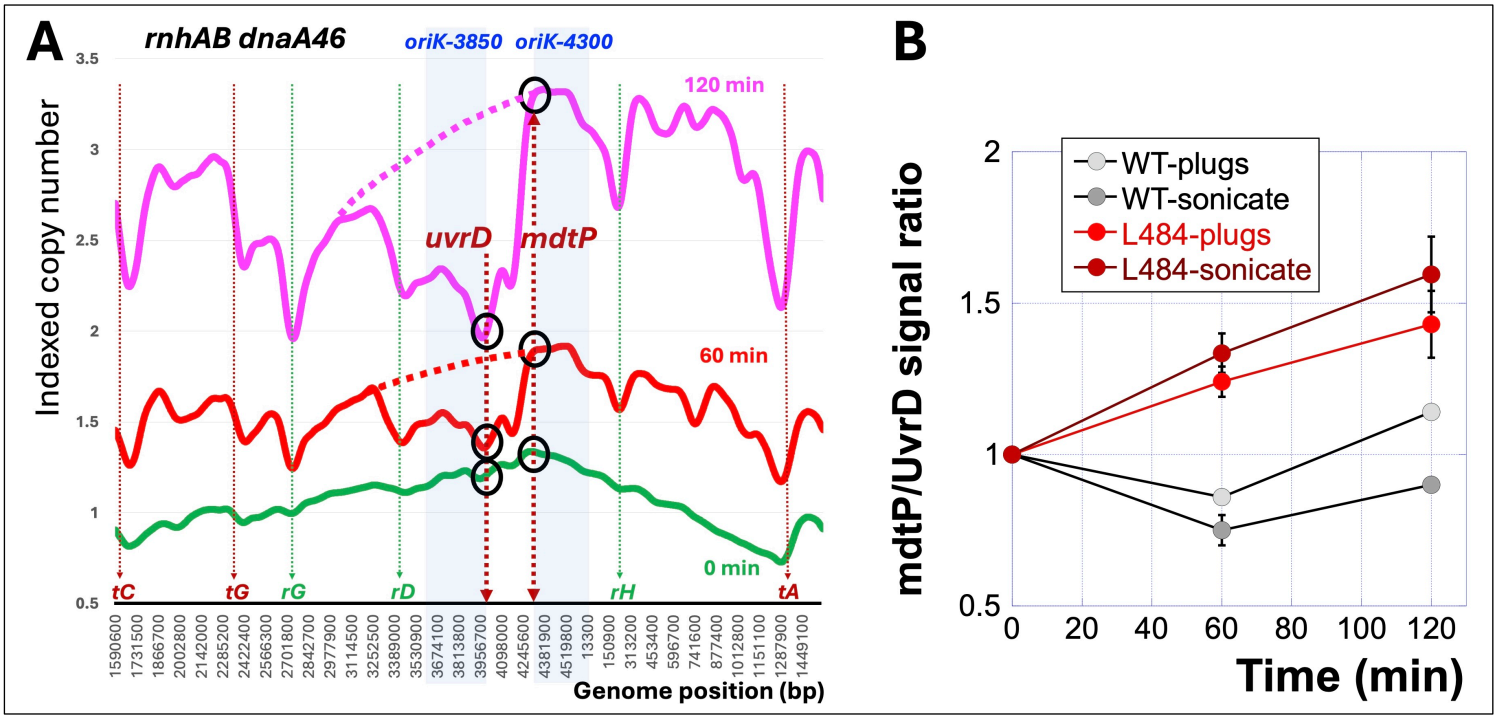

Fig. S14. Testing the reality of the *oriK-3850* absence in the *rnhAB dnaA*(Ts) mutant. Comparison of the mdtP/uvrD signal ratios in the WT (AB1157) and *rnhAB dnaA*(Ts) mutant (L-484) at 60 and 120 min post-UV relative to the signal ratio at 0 min. The *mdtP* gene (4,300) represents the top of the *oriK-4300* zone, while *uvrD* (4,000) represents the bottom of the nearby trough to the left.

A. The nested *rnhAB dnaA* profile with the two gene positions marked in brown and the compared profile values circled. Dashed lines connecting profile peaks at 60 and 120 min represent the suspected actual profiles, if DNA under-isolation indeed takes place.

B. The ratio of two gene DNA signals (by dot-blot hybridization), prepared by two different methods that maximize DNA isolation: direct transfer from agarose plugs (KOUZMINOVA AND KUZMINOV 2012) versus sonication of the SDS -cell lysates before phenol extraction (see Methods). The values are normalized to time =0.

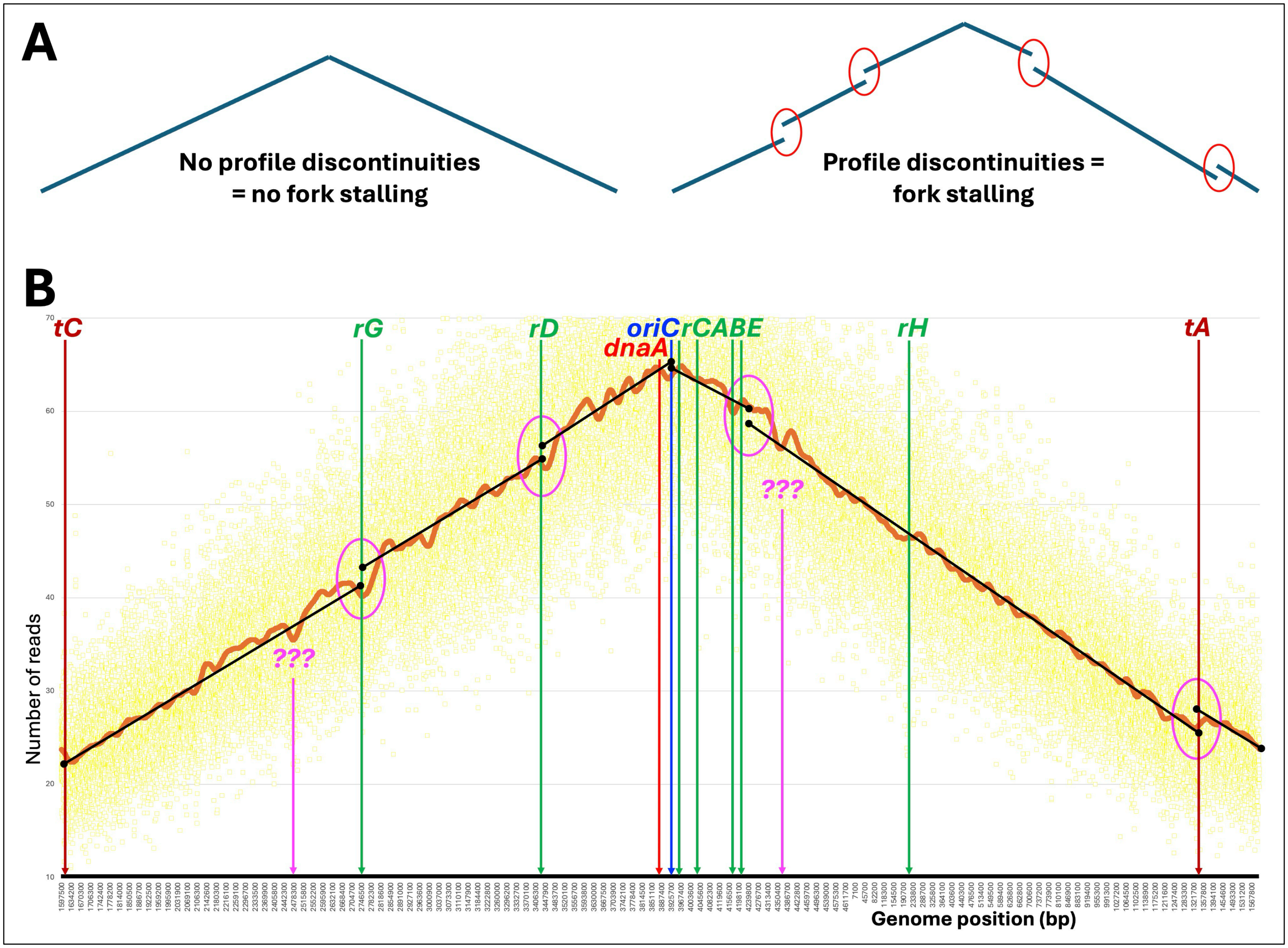

Fig. S15. Copy-number discontinuities in replication profile of growing wild type cells reflect replication-fork barriers. Strain AB1157, growth at 37°C.

A. A scheme of a replication profile lacking discontinuities of the average trendlines (left) and another profile with four discontinuities in the trendlines (besides the two expected ones at the origin and in the terminus).

B. In the WT profile (the same as in Fig. 1B top), average trendlines (black lines limited with dots) are drawn through the LOESS curve between the major discontinuities. A pink oval marks the position where the next trendline starts lower, meaning lowering of the copy number of the next segment and signaling replication impediment. The one on the right is the expected fork impediment at *terA*. The other three discontinuities either coincide with *rrn* operons or are caused by them (the *rrnCABE* cluster). There may be no discontinuity at *rrnH*, though.

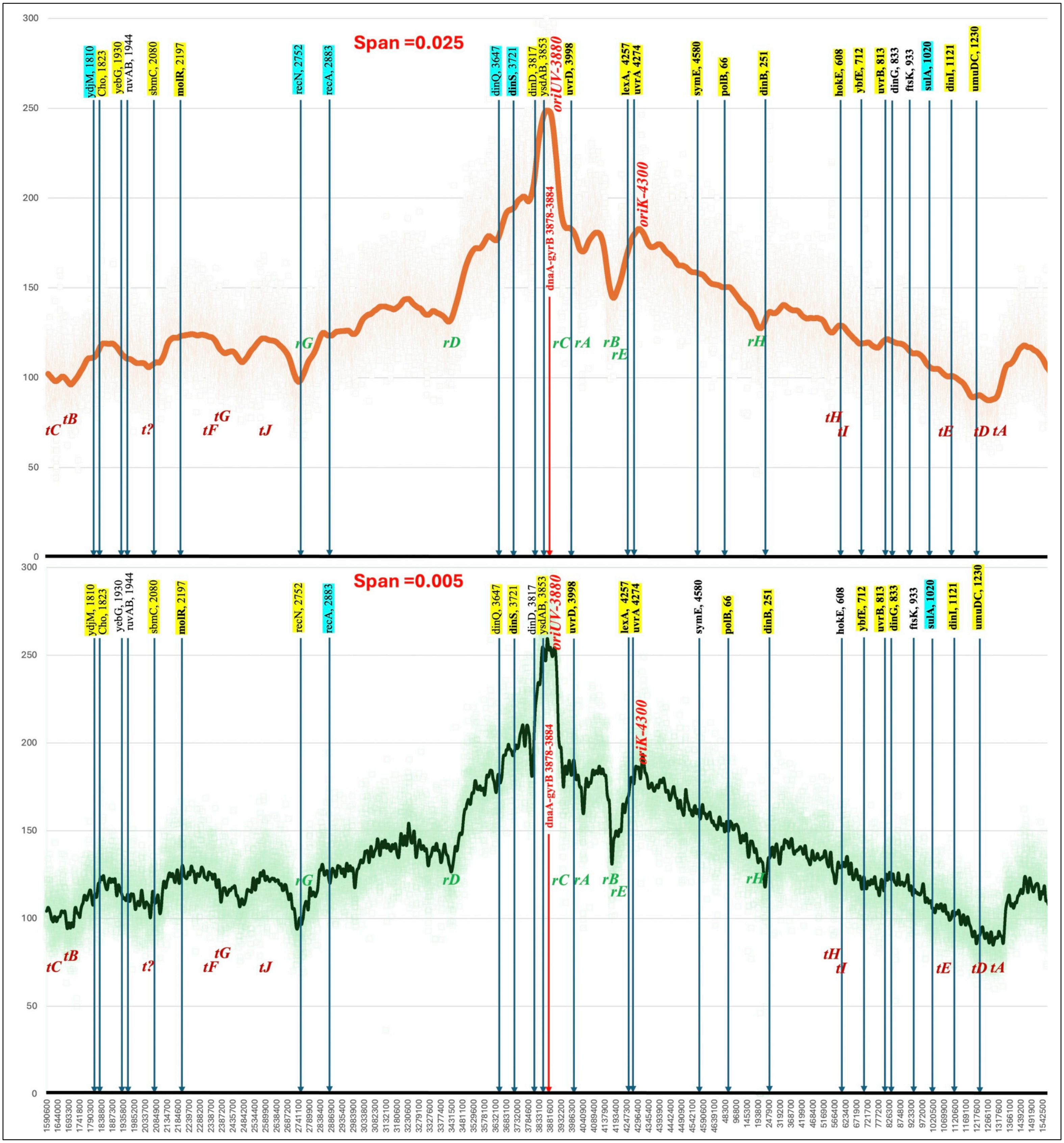

Fig. S16. Relationship between peaks and the SOS genes. The copy number profile of the *rnhAB* mutant 60 min post-UV is presented with two different smoothing LOESS parameters: span=0.025 (less detail) on the top, span=0.005 (more detail) on the bottom. Both profiles are marked with positions of *rrn* operons (green) and termination sites (brown), both underneath the

LOESS curves, as well as the two origins in red (*oriUV-3880* and *oriK-4300*). The SOS genes (at the top) are linked by vertical arrows with their chromosome coordinates (at the X-axis). The genes that are subjectively judged to coincide with local troughs of the profile are highlighted in cyan, those judged to coincide with local peaks are highlighted in yellow; the genes without highlights are judged to have no significant troughs or peaks nearby.

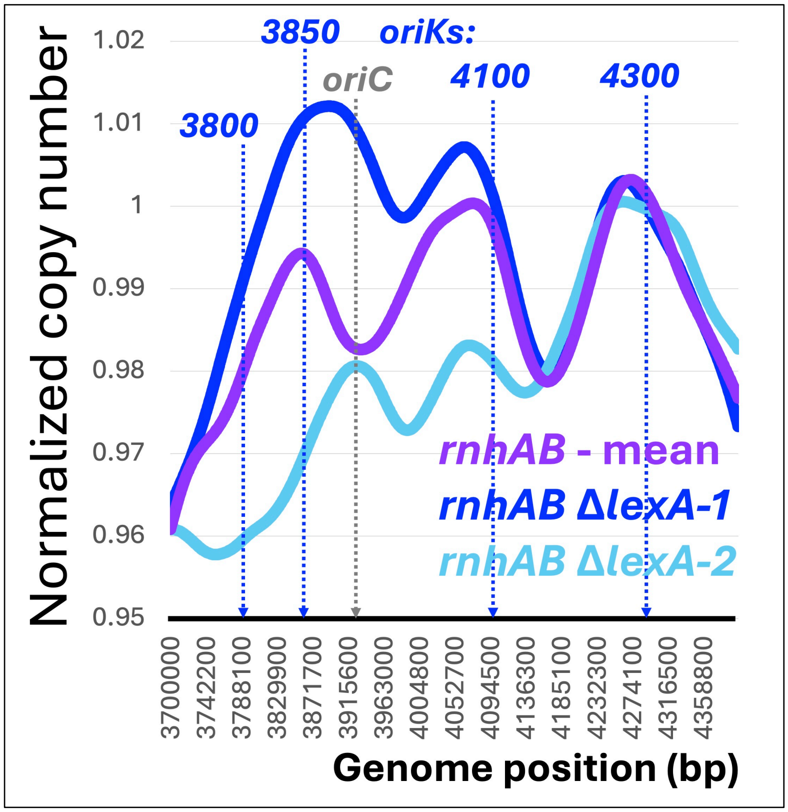

Fig. S17. *oriK-4300* is still functional in Δ*lexA* mutants.
The *lexA* gene was deleted with its own SOS-inducible promoter. The LOESS values in the origin macrodomain were normalized by the corresponding mean LOESS values of 50 kbp region centered at position 4,300 kb of the chromosome (like in Fig. 6E). Strains are L-416, L-564-1 and L-564-2.

Supplemental Tables

Table S1. Chromosomal coordinates (MG1655 vs AB1157) of potential replication-affecting loci and some genes. Positions of the *ter*-sites are from (MOREAU AND SCHAEFFER 2012). Colors match those of Fig. 1A: blue, *oriC*; green, *rrn* operons; brown, *ter*-sites. Additional colors: black, individual genes, yellow highlights — suspected new ter-sites; grey highlights — *ter*-sites showing no activity in the *rnhA* mutants.

Locus	Coordinates (kbp) MG1655	AB1157	
oriC	3926	same	
rrnC	3941	3942	
rrnA	4034	4036	
rrnB	4165	4167	
rrnE	4206	4208	
rrnH	223	224	
terH	599	600	
terI	625	same	
terE	1081	1082	
terD	1279	1280	
terA	1340	1342	
terC	1607	1609	
terB	1682	1684	
(terX-terK)	1975	1988	
(terY-terK)		2052	
terF	2316	2318	
terG	2375	2377	
terJ	2574	2576	
rrnG	2729	2731	
rrnD	3427	3429	
(oriC	3926	same)	— duplicated to close the chromosomal "circle"
(dnaA...gyrB)	3884-3878	same	
uvrD	4000	3998	
lexA	4257	same	
uvrA	4274	4271	
mdtP	4300	4301	

Table S2. Chromosomal coordinates of highest points in the *oriK* zones from Fig. 1C.

Zone #	position (Mb) Fig. 1C	Published positions	
		(MADUIKE *et al.* 2014)	(DIMUDE *et al.* 2015)
1	1.9	1.84-1.91	1.98
2	2.3	—	2.24
3	2.6	2.59-2.67	2.6
4	3.3	3.23	3.2
5	3.8	—	3.5
6	4.3	4.26-4.40	4.54
7	0.3	0.79-0.85	0.4
8	1.5	1.47-1.52	1.47

Table S3. *E. coli* strains.

Strain	Relevant genotype	Reference
**Published**		
AB1157*	Wild-type strain*	(BACHMANN 1987)
ER131	*ΔrnhA*::*cat*	(KOUZMINOVA et al. 2017)
SK129	*recB270*(Ts) *recC271*(Ts)	(KUSHNER 1974)
KKW58	Δ*thyA* Δ*deoCABD*	(KUONG AND KUZMINOV 2010)
ON152	*rnhA91*	(OGAWA AND OKAZAKI 1984)
L-27	*recA200(Ts)ΔsulA510*::*kan*	(KOUZMINOVA AND KUZMINOV 2004)
L-28	*ΔlexA609*::*cat recA441(Ts) sulA3*	(KOUZMINOVA AND KUZMINOV 2004)
L-404	*ΔrnhB782*::*kan*	(KOUZMINOVA et al. 2017)
L-413	*ΔrnhA*	(KOUZMINOVA et al. 2017)
L-416	*ΔrnhA ΔrnhB*	(KOUZMINOVA et al. 2017)

L-416-33	*ΔrnhA ΔrnhB rpoB*35 argE*::Tn*10*	(KOUZMINOVA AND KUZMINOV 2021)
L-476	*recB270*(Ts) *recC271*(Ts) *ΔrnhA*::*cat ΔrnhB782*::*kan*	(KOUZMINOVA et al. 2017)
L-483	*dnaA46*(Ts) *tna*::*Tn10 ΔrnhA*::*cat*	(KOUZMINOVA et al. 2017)
L-484	*dnaA46*(Ts) *tna*::*Tn10 ΔrnhA*::*cat ΔrnhB782*::*kan*	(KOUZMINOVA et al. 2017)

This study

L-183	*rnhA91 ΔoriC*::*cat*	ON152, deletion-replacement
L-192-2	*rnhA91 ΔoriC*::*cat oriCect -1023/2*	L-183 and integrated pEAK36
L-527	*thyA ΔrrnG(rrfG-rrsG)305*::*kan*	KK58, deletion-replacement
L-529	*thyA ΔrrnD(rrfF-rrsD)309*::*kan*	KK58, deletion-replacement
L-531-1	6*rrn*, *ΔrrnG(rrfG-rrsG)305*(::*FRT*)	AB1157 x P1 L-527, pCP20
L-531-2	5*rrn*, *ΔrrnG(rrfG-rrsG)305*(::*FRT*) *ΔrrnD(rrfF- rrsD)309*(::*FRT)*	L-531-1x P1 L-529, pCP20
L-532-1	*ΔrnhA ΔrrnG*	L-413 x P1 L-527, pCP20
L-532-2	*ΔrnhA ΔrrnG ΔrrnD*	L-532-1-x P1 L-529, pCP20
L-534-1	6rrn, *ΔrnhA ΔrnhB ΔrrnG::(rrfG-rrsG)305(::FRT)*	L-416 x P1 L-527, pCP20
L-534-2	5rrn*, ΔrnhA ΔrnhB ΔrrnG(yfiS,rrfG-rrsG)305*(::*FRT*) *ΔrrnD(rrfF-rrsD)309(::FRT)*	L-534-1x P1 L-529, pCP20
L-543	*ΔrrnH(rrsH-aspU)315*::*cat*	MG1655, deletion-replacement
L-544	*ΔrrnH(rrsH-aspU)315*::*cat ΔrnhB782*::*kan*	L-543 x P1 L-404
L-545	*ΔrnhA*	MG1655 x P1 ER131, pCP20
L-548	*ΔrnhA ΔrrnH (rrsH-aspU)315*::*cat ΔrnhB782*::*kan*	L-545 x P1 L-544
L-554	4rrn*, ΔrrnG ΔrrnD ΔrrnH*::*cat ΔrnhA ΔrnhB782*::*kan*	L-532-2 x P1 L-548
L-555	*ΔrnhA ΔoriC*::*cat*	L-413 x P1 L-192-2 pEAK39
L-556	*ΔrnhA ΔrnhB ΔoriC*::*cat*	L-416 x P1 L-192-2 pEAK39

L-559	*ΔlexApr::kan recA441(ts) sulA3*	L-28, deletion-replacement
L-562	*ΔsulA::kan ΔrnhA ΔrnhB*	L-416 x P1 L-27
L-563	*ΔsulA ΔrnhA ΔrnhB*	L-562, pCP20
L-564-1	*ΔsulA ΔlexApr*::kan *ΔrnhA ΔrnhB*	L-563 x P1 L-559
L-564-2	*ΔsulA ΔlexApr*::kan *ΔrnhA ΔrnhB*	L-563 x P1 L-559

* — complete genotype of AB1157 includes: F– lambda– rac- *thi-1 hisG4 Δ(gpt-proA)62 argE3 thr-1 leuB6 kdgK51 rfbD1 araC14 lacY1 galK2 xylA5 mtl-1 tsx-33 glnV44 rpsL31*

Table S4. Plasmids used in the study

**Plasmid**	**Replicon/drug resistance/other genes**	**Reference**
pCP20		
pEAK36	*oriC* / *kan* / “NotI 20”	this study
pEAK39	pSC101 / *aadA* / *rnhA*	(KOUZMINOVA et al. 2017)

pEAK36-minichromosome construction: 2011 bp PCR fragment containing oriC was amplified with the primers #65, # 66 positions:3922382- 3922400, 3924391- 3924373 (described as ori primers in Kouzminova, 2008) and cloned into pCR2.1-TOPO vector to produce pEAK23-1. Then the fragment was cleaved out with *Xba*I and *Spe*I and ligated with 1.3 kbp XbaI fragment from pK57 carrying the *neo* gene to give rise to pEAK31- minichromosome. The 2019 pb fragment "NotI 20" was amplified by PCR from chromosomal template with primers #141, 142 with positions: 304355-304373, 306356-306374 (described as N20 primers in (KOUZMINOVA AND KUZMINOV 2008)) and was subcloned into pCR2.1-TOPO vector to give rise to pEAK34. Then the "NotI 20" fragment was cut out of pEAK34 with *Not*I and *Spe*I and ligated with pEAK31-minichromosome vector, linearized with *Xba*I and *Not*I, to give rise to pEAK36-minichromosome.

L-192-2 construction: pEAK36-minichomosome integration into the chromosome of the strain L-183 (*rnhA91 ΔoriC*::*cat*) resulted from recombination of the homologous "NotI 20" sequences. Kanamycin resistant colonies containing ectopic *oriC* integrated into the chromosome were identified as larger size colonies (compare to the colonies of L-183 host) at 42°C on LB-agar plates.

Table S5. Primers

Primers for *ΔoriC*

#159. 5'-TTTTCCATCCCCTGCATAGAGCGGACGATTTGCATCTGCA
-TGTAGGCTGGAGCTGCTTCG-3'
#160. 5'-CCGAAGCAGCTCGGTTATGACGTAGGCTGCTTTATCGGCA
-CATATGAATATCCTCCTTAG-3'
#161. 5'-CCATCCCCTGCATAGAGCGG-3'
#162. 5'-GGGCGCGTATTGATGTCAGC-3'

Primers for *ΔrrnG*

#305. 5'-TTGTTGCATATCATTATGCAACCTTAACCATGAATTTAGT- GTGTAGGCTGGAGCTGCTTC -3'
#306. 5'-GATAAAACGAGCCCTTCGGGGCTCGTTTTTGTCTATAAGT-
ATTCCGGGGATCCGTCGACC-3'
#307. 5'-CCGTTACAGTACTTTCAGCCATGC-3'
#308. 5'-AGACCGGATTGTCGCCGTCC-3'

Primers for *ΔrrnD*:

#309. 5'-CGCAGGTAATCCATTAATTGAATGTTAGTTCGAAAAGCAA- GTGTAGGCTGGAGCTGCTTC

#310. 5’-ACTTTGGGGGCATTATTGGCCTTGTGCAAGTCTTTTAGTA- ATTCCGGGGATCCGTCGACC 3’

#311. 5’-GCGCATCCTAAATCAGAGCG-3’

#312. 5’-ACGCGCTGACCGATTTGTGG-3’

Primers for *ΔrrnH*

#315. 5’-tttttccgcttgtcttcctgagccgactccctataatgcgTGT AGG CTG GAG CTG CTT CG-3’
#316. 5’-cgtaaaatgcagaggatttttgcgattctggcaataatagCAT ATG AAT ATC CTC CTT AG-3’
#317. 5’-cagacctgccgcaagcgat-3’
#318. 5’-aaccaaatgcagggatagcc-3’

Primers for rrnG probes:

#327 5’-caggtatcggcaatcagtcattcg-3’
#328 5’-actcttcaggcagttaaatgggc-3’
#329. 5’-caccacctaatcctccaccag-3’
#330. 5’-catggctgaaagtactgtaacgg-3’
#331. 5’-ttcgatctgctgctgaattgcac-3’
#332. 5’-acaactgaaggccgcttttcc-3’
#333. 5’-aaagcggccttcagttgttcc-3’
#334. 5’-cgatatcgtactcgccaatacgc-3’

Primers for mdtP probe:
#346. 5’-CGTCAACTTTC ACGTCTGCTG-3’
#347. 5’-ATCCGGACCGTACTTCTCTTTG-3’

Primers for uvrD probe:
#292. 5’-CGATCTGATGCAAACCTGGG-3’
#293. 5’-TTAGGCCAAATAAGGTGCGC-3’

Primers for *ΔlexApr*::*kan*
#334. 5’-<u>ATTACCTG GTGCATTCTG TTATGGTCGCATTTTGGATAAC</u> TGTAGGCTGGAG CTGCTT CG-3’

#335. 5’-<u>AAACCGCGACGCCAGGCGGCATCGCGGTCTCAGAGATATG</u> CATATG AATATCCTCCTTAG

References

Bachmann, B. J., 1987 Derivations and genotypes of some mutant derivatives of *Escherichia coli* K-12, pp. 1190-1219 in *Escherichia coli and Salmonella typhimurium. Cellular and Molecular Biology*, edited by F. C. Neidhardt. American Society for Microbiology, Washington, D.C.

Condon, C., S. French, C. Squires and C. L. Squires, 1993 Depletion of functional ribosomal RNA operons in *Escherichia coli* causes increased expression of the remaining intact copies EMBO J. 12**:** 4305-4315.

Dimude, J. U., A. Stockum, S. L. Midgley-Smith, A. L. Upton, H. A. Foster *et al.*, 2015 The Consequences of Replicating in the Wrong Orientation: Bacterial Chromosome Duplication without an Active Replication Origin. MBio 6**:** e01294-01215.

Kouzminova, E. A., F. F. Kadyrov and A. Kuzminov, 2017 RNase HII Saves *rnhA* Mutant *Escherichia coli* from R-Loop-Associated Chromosomal Fragmentation. J. Mol. BIol. 429**:** 2873-2894.

Kouzminova, E. A., and A. Kuzminov, 2004 Chromosomal fragmentation in dUTPase-deficient mutants of *Escherichia coli* and its recombinational repair. Mol. Microbiol. 51**:** 1279-1295.

Kouzminova, E. A., and A. Kuzminov, 2008 Patterns of chromosomal fragmentation due to uracil-DNA incorporation reveal a novel mechanism of replication-dependent double-strand breaks. Mol. Microbiol. 68**:** 202-215.

Kouzminova, E. A., and A. Kuzminov, 2012 Chromosome demise in the wake of ligase-deficient replication. Mol. Micorbiol. 84**:** 1079-1096.

Kouzminova, E. A., and A. Kuzminov, 2021 Ultraviolet-induced RNA:DNA hybrids interfere with chromosomal DNA synthesis. Nucleic Acids Res. 49**:** 3888-3906.

Kuong, K. J., and A. Kuzminov, 2010 Stalled replication fork repair and misrepair during thymineless death in *Escherichia coli*. Genes Cells 15**:** 619-634.

Kushner, S. R., 1974 In vivo studies of temperature-sensitive *recB* and *recC* mutants. J. Bacteriol. 120**:** 1213-1218.

Maduike, N. Z., A. K. Tehranchi, J. D. Wang and K. N. Kreuzer, 2014 Replication of the *Escherichia coli* chromosome in RNase HI-deficient cells: multiple initiation regions and fork dynamics. Mol. Microbiol. 91**:** 39-56.

Moreau, M. J., and P. M. Schaeffer, 2012 Differential Tus-Ter binding and lock formation: implications for DNA replication termination in *Escherichia coli* Mol. Biosyst. 8**:** 2783-2791.

Ogawa, T., and T. Okazaki, 1984 Function of RNase H in DNA replication revealed by RNase H defective mutants of *Escherichia coli*. Mol. Gen. Genet. 193**:** 231-237.
